# Nuclear Hormone Receptor Regulation of MicroRNAs Controls Innate Immune Responses in *C. elegans*

**DOI:** 10.1371/journal.ppat.1003545

**Published:** 2013-08-22

**Authors:** Feng Liu, Chen-Xi He, Li-Jun Luo, Quan-Li Zou, Yong-Xu Zhao, Ratni Saini, San-Feng Han, Hans-Joachim Knölker, Li-Shun Wang, Bao-Xue Ge

**Affiliations:** 1 Institute of Health Sciences, Shanghai Institutes for Biological Sciences, Chinese Academy of Sciences/Shanghai JiaoTong University School of Medicine, Shanghai, China; 2 Graduate School of Chinese Academy of Sciences, Beijing, China; 3 Shanghai Key Laboratory of Tuberculosis, Clinical and Translational Research Center, Shanghai Pulmonary Hospital, Tongji University School of Medicine, Shanghai, China; 4 Department of Chemistry, Technical University of Dresden, Dresden, Germany; 5 Key Laboratory of Cell Differentiation and Apoptosis of Chinese Ministry of Education, Shanghai Jiao-Tong University School of Medicine, Shanghai, China; Massachusetts General Hospital, Harvard Medical School, United States of America

## Abstract

Nuclear hormone receptors respond to small molecules such as retinoids or steroids and regulate development. Signaling in the conserved p38/PMK-1 MAP kinase pathway regulates innate immunity. In this study, we show that the *Caenorhabditis elegans* nuclear receptor DAF-12 negatively regulates the defense against pathogens via the downstream *let-7* family of microRNAs, which directly target SKN-1, a gene downstream of PMK-1. These findings identify nuclear hormone receptors as components of innate immunity that crosstalk with the p38/PMK-1 MAP kinase pathway.

## Author Summary

When infected by the *Pseudomonas aeruginosa*, the nematode *Caenorhabditis elegans* invokes an innate immune response that protects the worm from pathogenic attack. The appropriate level of immune response in *C. elegans* requires the accurate regulation of multiple signal pathways, especially signals of repression, which attenuate the expression of pathogen-responsive genes. In the current study, we identified the nuclear hormone receptor DAF-12 and its downstream *let-7* family of microRNAs, *mir-84 and mir-241*, are required for the regulation of *C. elegans* innate immunity against *P. aeruginosa* infection. Dafachronic acids, as DAF-12 ligands, can dramatically suppress the resistance of *C. elegans* to *P. aeruginosa* infection. Inhibition of the conserved PMK-1/p38 MAP kinase pathway can markedly attenuate the promoted resistance of *daf-12* and *let-7* family of microRNAs mutants to *P. aureginosa* infection. However, neither *daf-12* nor *let-7* family of microRNAs affect the activation of PMK-1/p38. Moreover, our data also reveals the role of SKN-1 in integrating the signals from the PMK-1/p38 MAPK and DAF-12-*let-7*s pathways to mediate the *C. elegans* innate immune response.

## Introduction

Innate immunity is an evolutionarily conserved response to pathogens and forms the first line of defense for most organisms. When infected by pathogens, the nematode *Caenorhabditis elegans* mounts a rapid innate immune response and produces an array of anti-microbial genes, similar to other organisms throughout the animal kingdom [1], [2]. Several conserved signaling pathways that function in the perception of and defense against bacterial pathogens have been identified in *C. elegans*. These pathways include the NSY-1/PMK-1 MAP kinase signaling pathway, the DAF-2/DAF-16 insulin/insulin-like growth factor (IGF)-1 like signaling pathway, the DBL-1/transforming growth factor-β (TGF-β) signaling pathway and the BAR-1 β-catenin signaling pathway [1]–[4]. Although many conserved innate immune components have been identified in *C. elegans* using genetic and biochemical approaches, extensive characterization of the signaling networks that regulate the host response and outcome of infections is warranted.

Nuclear hormone receptors (NRs) are a class of transcription factors that are regulated by small lipophilic hormones. In all, 284 NRs have been identified in *C. elegans*, and approximately 20 of them have been genetically analyzed [5]. The dauer formation abnormal gene *daf-12*, a well-characterized nuclear hormone receptor, and the orphan receptors *nhr-8* and *nhr-48* are the conserved homologs of the mammalian vitamin D receptor and liver-X receptor [6], [7]. DAF-12 regulates developmental progression and arrest in response to environmental cues [6], [8]. In favorable conditions, the activation of TGF-β and insulin/IGF-1 signaling cascades results in the production of the DAF-12 steroidal ligands, dafachronic acids (DAs). DAs are synthesized from cholesterol via a multi-step pathway involving the daf-36 Rieske-like oxygenase and the daf-9 cytochrome P450 enzyme, which promote a rapid progression through four larval stages (L1 to L4) to reproductive adults [9]–[14]. In unfavorable environments, DAs expression is suppressed, and DAF-12, without its ligand, binds to the co-repressor DIN-1, resulting in an arrest at a stress-resistant, long-lived alternative third larval stage, called the dauer diapauses (L3d) [15]. In addition, DAF-12 regulates the normal lifespan of worms and the longevity of germline-ablated animals [16]–[21]. However, the role of DAF-12 in the immune regulation of *C. elegans* remains unknown.

MicroRNAs (miRNAs) are small non-coding RNA molecules that repress target gene expression by base-pairing with partially complementary sequences in the 3′-untranslated regions (3′-UTR) of target mRNAs [22], [23]. MiRNAs influence molecular signaling pathways and regulate many biological processes, including immune function [24]. Originally discovered in *C. elegans*, lethal-7 (*let-7*) miRNA is conserved across species in both sequence and temporal expression [25], [26]. In *C. elegans*, the *let-7* miRNA homologs *mir-48*, *mir-84* and *mir-241* (together referred to as *let-7*s) regulate developmental timing and promote cellular differentiation pathways [27], [28]. The human *let-7*-related miRNAs also have anti-proliferative functions, and the downregulation of *let-7* levels is associated with a variety of cancers, such as lung, breast and colon cancer [27], [28]. DAF-12 and its steroidal ligands activate the expression of *let-7s*, which downregulate the heterochronic gene *hbl-1*, thus integrating environmental signals and developmental progression [29], [30]. However, the functional role of *let-7* family of miRNAs in the innate immune responses to pathogens is largely unknown. Hence, we sought to investigate whether DAF-12 and the *let-7* family of miRNAs play a role in the regulation of the innate immune responses to bacterial infection in *C. elegans*.

## Results

### DAF-12 regulates pathogenic defense

**Figure 1:**
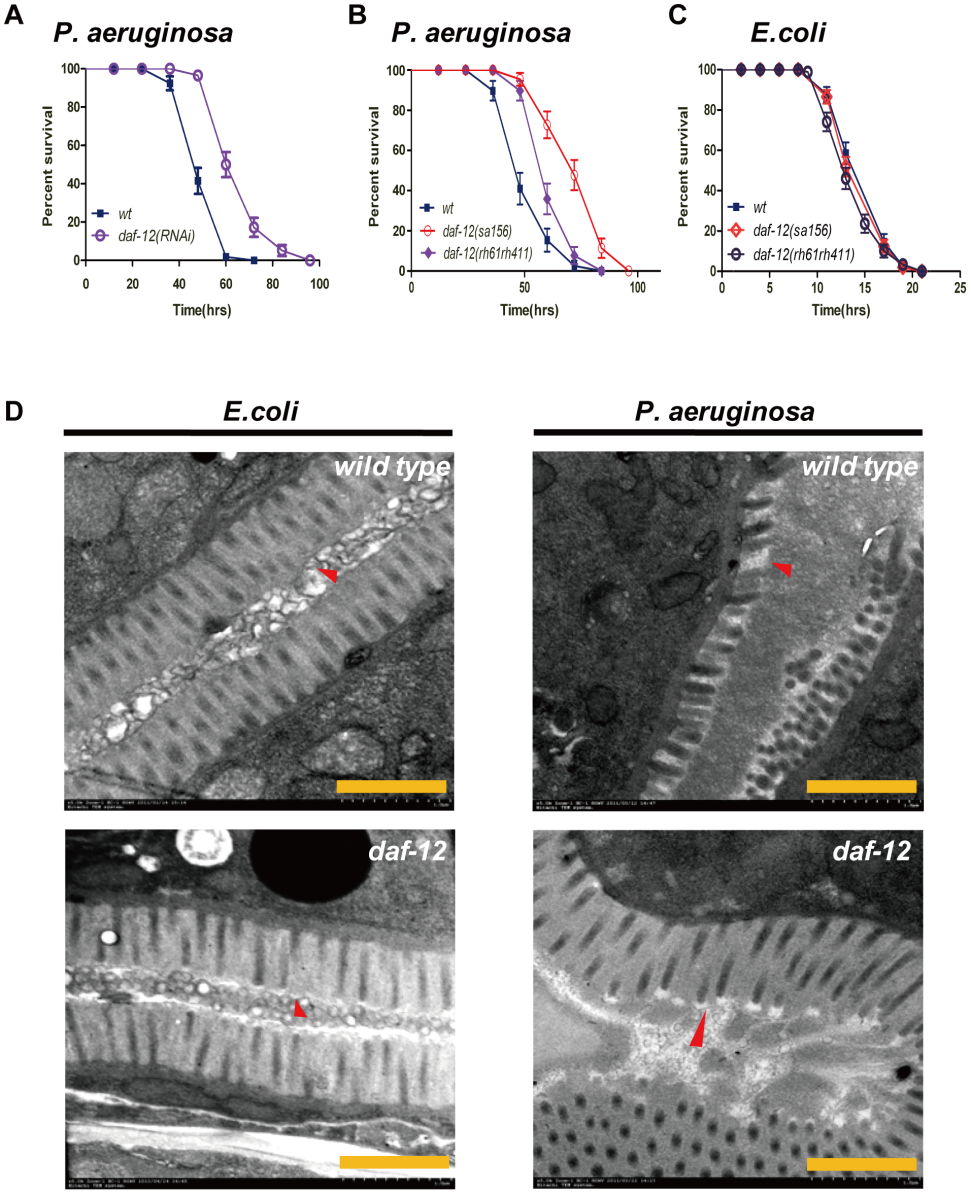
DAF-12 regulates pathogenic defense. (**A**) Survival curve of *daf-12* RNAi-treated worms exposed to *P. aeruginosa* (PA) relative to control RNAi-treated worms (*P*<0.0001). (**B**) *P. aeruginosa* killing assay of wild-type N2, *daf-12*(*sa156*) (*P*<0.001) and *daf-12* (*rh61rh411*) (*P*<0.001) worms. (**C**) Lifespan curve of N2, *daf-12*(*sa156*) (*P* = 0.4118) and *daf-12*(*rh61rh411*) (*P* = 0.0019) worms. (**D**) Transmission electron micrographs (TEM) of a gut section from wild-type N2 and *daf-12(sa156)* worms fed *E. coli* or *P. aeruginosa* for 48 hours. Red arrows indicate microvillis. (Scale bars 1000 nm) All data shown above are representative of at least three independent experiments (n≥50 adult nematodes per strain).

We used an RNAi feeding method to search for the host components that influence the response of *C. elegans* to infection with *Pseudomonas aeruginosa* strain PA14, which is a human opportunistic pathogen that can also infect and kill *C. elegans*. Using 399 RNAi clones targeting transcription factors, we identified 17 transcriptional factors that affect the survival of worms on the *P. aeruginosa* lawns (**Table S1**). Among these candidates, treatment with *daf-12* RNAi improved either the resistance of *C. elegans* to *P. aeruginosa* infection or its survival on an avirulent *E. coli* lawn (**Fig. 1A** **, Fig. S1A**). Transgenic *daf-12(dhls26)* worms containing *daf-12::GFP* were more susceptible to *P. aeruginosa* (**Fig. S1B**). DAF-12, along with NHR-8 and NHR-48, is a conserved homolog of the mammalian vitamin D/liver X receptor (LXR) in *C. elegans* [6], [7]. However, inhibition of *nhr-8* and *nhr-48* increased pathogenic susceptibility to *P. aeruginosa* infection (**Fig. S2A**), suggesting that *nhr-8* and *nhr-48* have roles opposite to that of *daf-12* in innate immune regulation.

To further investigate the role of DAF-12 in the immune response to bacterial infection, we examined the survival rate and lifespan of *daf-12* alleles that have been previously identified on the basis of development and aging [6], [8]. A *daf-12* null mutant *daf-12*(*rh61rh411*) that contained two nonsense mutations affecting both DNA binding domain (DBD) and ligand binding domain (LBD) [6] and was more resistant to *P. aeruginosa*, had a shortened lifespan compared to wild-type N2 animals (**Fig. 1B and 1C**). The *daf-12(sa156)* mutant containing a C121Y mutation in the zinc finger of DBD [6], which may interrupt the DNA binding activity of DAF-12, displayed a normal lifespan but increased resistance to *P. aeruginosa* infection (**Fig. 1B and 1C**). In contrast, the two other two mutants, *daf-12(m20)*, which has a nonsense mutation affecting DBD [6], and *daf-12(m25)*, containing a M562I mutation in LBD [6], exhibited extended lifespans and normal pathogenic resistance to *P. aeruginosa* infection (**Fig. S3A and 3B**). These results not only identify DAF-12 as a negative regulator of innate immune responses to the infection of *P. aeruginosa* but also suggest a cross-talk between developmental progression and host defense.

We have also examined whether DAF-12 is involved in *C. elegans* host defense to other different pathogens, and found that inhibition of *daf-12* greatly increased the resistance of *daf-12(sa156)* mutants to *Staphyloccocus aureus* infection (**Fig. S2B**). We next performed transmission electron microscopy analysis to examine gut cells of wild-type worms or *daf-12*(*sa156*) worms fed *P. aeruginosa* or *E. coli*. When fed *E. coli*, both of the wild-type N2 and *daf-12(sa156)* worms display normal intestinal ultrastructure, whereas when infected by *P. aeruginosa*, the *daf-12*(*sa156*) worms exhibited less severely damaged gut cells and more intact microvilli than in the wild-type worms (**Fig. 1D**). To determine the cellular localization of DAF-12, we utilized previously generated transgenic *daf-12(dhls26)* worms containing *daf-12::GFP* [7] and showed a significant accumulation of DAF-12 in the nuclei of neurons and intestinal cells when worms were fed *E. coli*. However, when infected with *P. aeruginosa*, DAF-12 expression was not affected (**Fig. S4C**), but the associated GFP signal was diffusely distributed throughout both neuronal and intestinal cells (**Fig. S4A and S4B**), suggesting that the *P. aeruginosa* infection suppresses nuclear localization of DAF-12 and promotes its translocation to the cytoplasm.

### DAF-12 regulates antimicrobial genes expression

**Figure 2:**
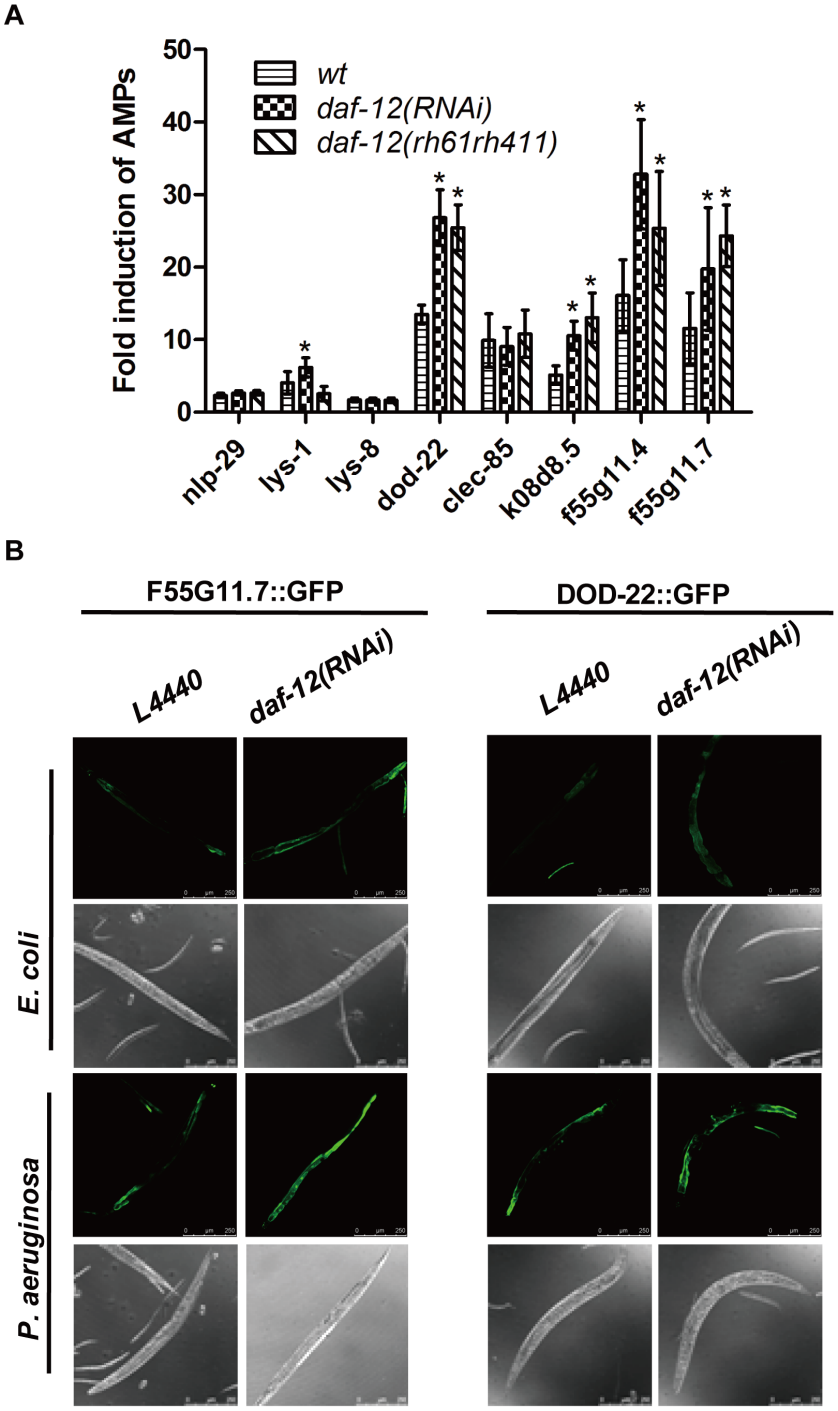
DAF-12 regulates antimicrobial gene expression. (**A**) qRT-PCR analysis of anti-microbial peptide (AMP) gene expression in N2, *daf-12*(RNAi) and *daf-12*(*rh61rh411*) worms infected with *P. aeruginosa* for 24 hours. The data shown are the mean ± SEM of three independent experiments, each of which was performed in triplicate, *P<0.05. (**B**) Confocal microscopy of *daf-12* RNAi-treated or control RNAi-treated *dod-22::GFP* and *F55G11.7::GFP* worms on *E. coli* or *P. aeruginosa* for 24 hours. The data are representative of three independent experiments (n≥50 adult nematodes per strain).

We then examined the effect of *P. aeruginosa* infection on the expression of eight selected anti-microbial genes that are regulated by the NSY-1/PMK-1 pathway or the insulin/IGF-1-like pathway [31]. We found that in the *daf-12*(*rh61rh411*) and *daf-12* RNAi-treated worms infected with *P. aeruginosa*, expression levels of five of the eight anti-microbial genes were significantly higher compared to the wild-type control (**Fig. 2A**). To further confirm the quantitative RT-PCR results, we treated the *dod-22::gfp* or *F55G11.7::gfp* transgenic worms with *daf-12* RNAi, fed them *E. coli* or *P. aeruginosa*, and then subjected them to confocal image analysis. Treatment with *daf-12* RNAi greatly increased the expression of dod-22::GFP and F55G11.7::GFP at both basal *E. coli* levels and in *P. aeruginosa*-induced levels (**Fig. 2B** **, Fig. S5A and S5B**).

### Dafachronic acids (DAs) regulate pathogenic defense via DAF-12

**Figure 3:**
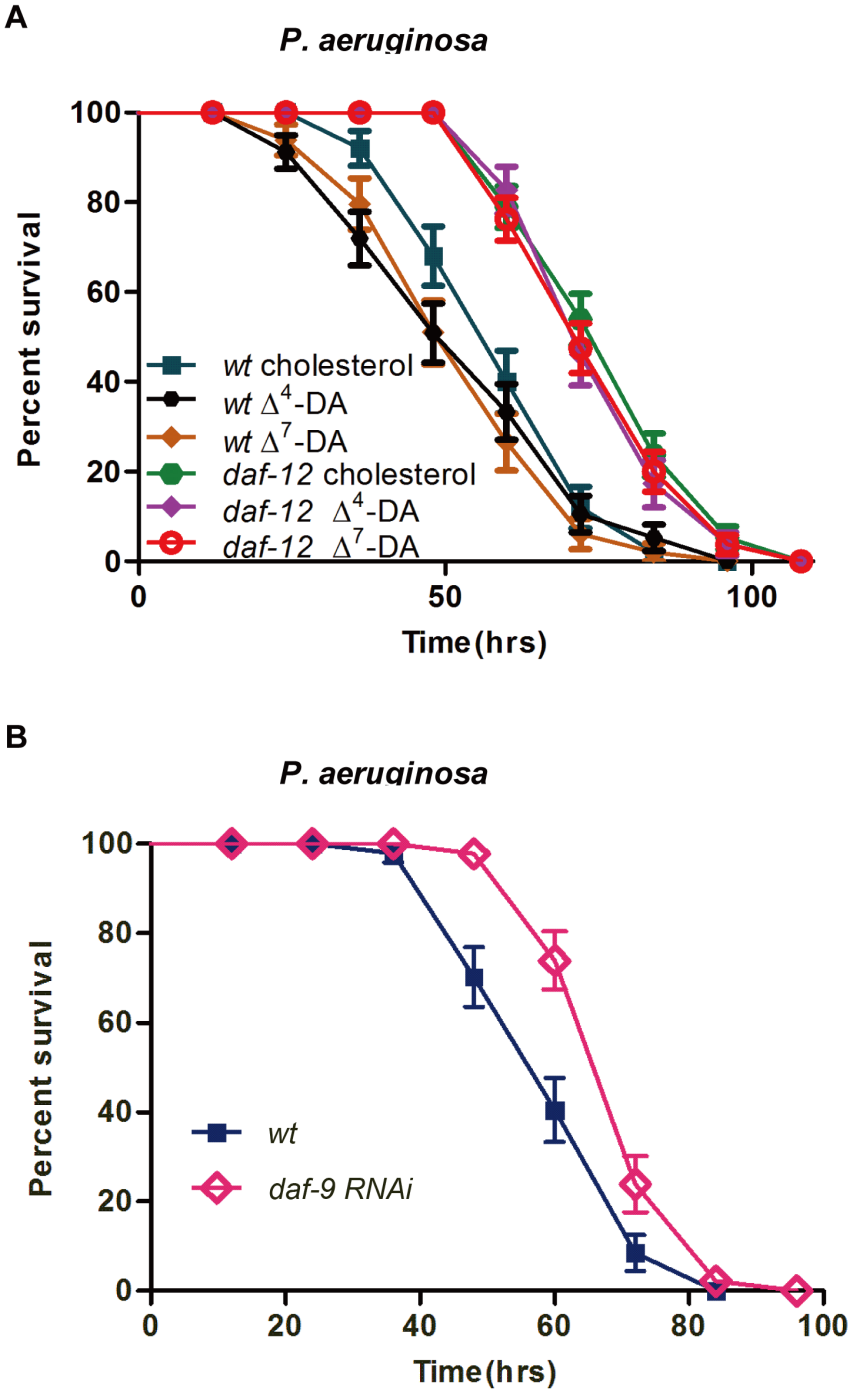
Dafachronic acids (DAs) regulate pathogenic defense through DAF-12. (**A**) *P. aeruginosa* killing of N2 and *daf-12(sa156)* worms grown with cholesterol (400 nM), Δ^4^-DAs (400 nM) (*P* = 0.2845 and 0.4552, respectively) and Δ^7^-DAs (400 nM) (*P* = 0.0426 and 0.5508, respectively). (**B**) *P. aeruginosa* killing assay of *daf-9 RNAi (P<0.001)* animals. All data shown are representative of at least three independent experiments (n≥50 adult nematodes per strain).

DAF-12 is known to control *C. elegans* response to its environment. Under favorable conditions, the stimulation of the insulin/IGF-1 andTGF-β pathways leads to the production of sterol-derived dafachronic acids (DAs). Δ^4^-DA and Δ^7^-DA bind to DAF-12, leading to developmental progression [10], [11]. The substitution of dietary cholesterol with Δ^7^-DAs reduced the resistance of wild-type worms, but not *daf-12*(*sa156*) worms, to *P. aeruginosa* infection (**Fig. 3A**). An increased dose of Δ^7^-DA did not lead to further increases in pathogenic susceptibility of the wild-type N2 worm to *P. aeruginosa* infection (**Fig. S6**). DAs are derivatives of dietary cholesterols that are synthesized via several pathways involving the cytochrome P-450 DAF-9 and the SAM-dependent methyltransferase STRM-1 [12], [13], [32]. Inhibition of DAF-9 expression by RNAi feeding increased the resistance of the worm to *P. aeruginosa* infection (**Fig. 3B**). In unfavorable environments, the downregulation of the insulin/IGF-1 and TGF-β pathways suppresses DA production, and without its ligand, DAF-12 associates with the co-repressor DIN-1 to promote dauer programs [15]. Thus, we next examined the role of DIN-1, a co-repressor of DAF-12, in the immune response of *C. elegans*. The inhibition of *din-1* did not affect the survival of wild-type worms on a *P. aeruginosa* lawn, but moderately attenuated the extended pathogenic resistance of *daf-12* RNAi worms (**Fig. S7A**), suggesting that DAF-12 regulating the immune response of *C. elegans* may be partially dependent on *din-*1.

### Inhibition of DAF-12-mediated immunity by NSY-1/PMK-1

**Figure 4:**
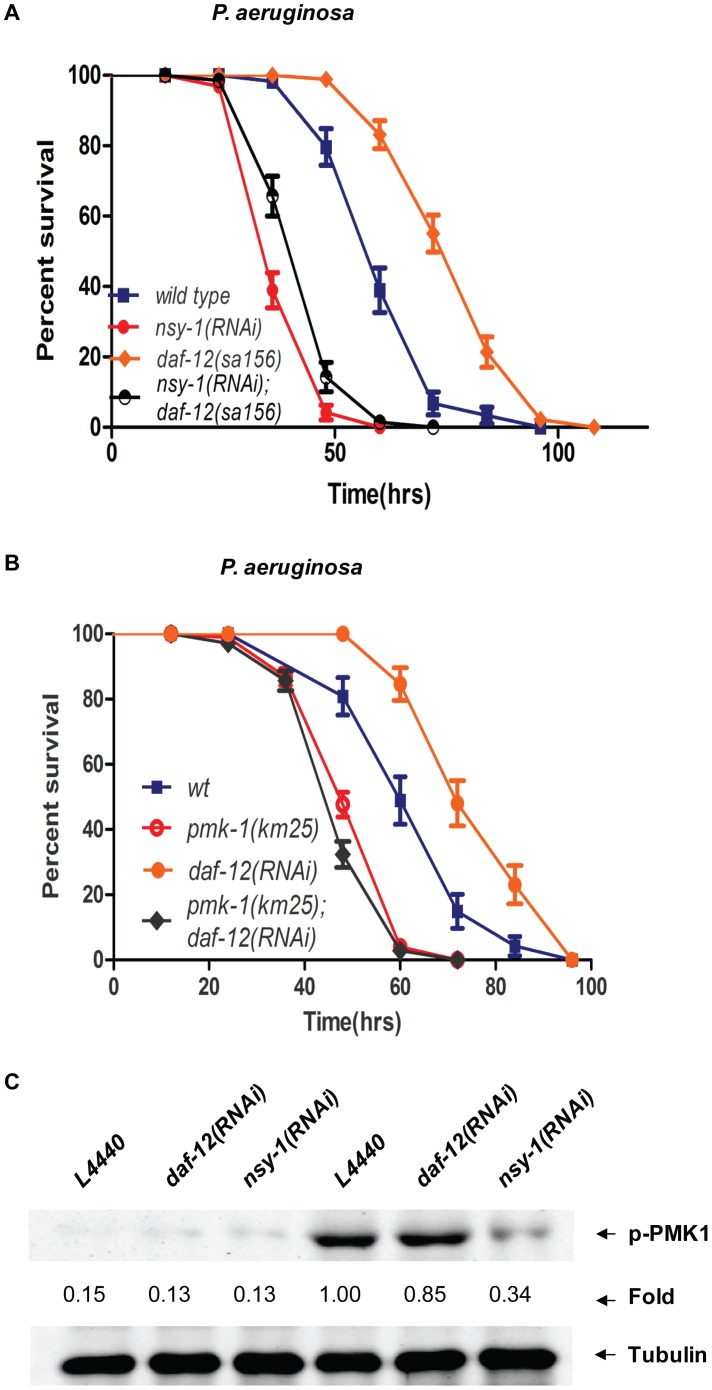
Inhibition of DAF-12-mediated immunity by NSY-1/PMK-1. (**A**) Survival curve of wild-type N2, *daf-12*(*sa156*) (*P*<0.0001), *nsy-1*(RNAi) (*P*<0.0001) and *daf-12*(*sa156*);*nsy-1*(RNAi) (*P* = 0.003) worms exposed to *P. aeruginosa*. (**B**) Survival curve of N2, *daf-12*(RNAi) (*P*<0.0001), *pmk-1*(*km25*) (*P*<0.0001) and *pmk-1*(km25);*daf-12*(RNAi) (*P* = 0.02) worms on *P. aeruginosa*. (**C**) Immunoblot analysis using anti-phospho-p38 antibody and anti-tubulin antibody (loading control) of the lysates from N2, *nsy-1*(RNAi) and *daf-12*(RNAi) young adults fed *E. coli* or *P. aeruginosa*. All data shown are representative of at least three independent experiments (n≥50 adult nematodes per strain).

Several conserved signaling pathways, including the NSY-1/PMK-1 pathway and the insulin/IGF-1-like pathway, are involved in the pathogenic defense of *C. elegans* [2]. The loss of function of the insulin receptor DAF-2 activates the downstream target DAF-16, which triggers the expression of anti-microbial genes in response to pathogenic infection [3], [33]. However, *daf-16* RNAi had no effect on the prolonged survival of *daf-12*(*sa156*) worms infected with *P. aeruginosa* (**Fig. S7B**). We then tested whether the NSY-1/PMK-1 pathway is involved in the enhanced resistance of *daf-12* mutants to *P. aeruginosa*. Either inhibition of *nsy-1* by RNAi or mutation of *pmk-1* attenuated the enhanced pathogenic resistance of *daf-12(sa156)* worms or *daf-12* RNAi-treated worms, respectively (**Fig. 4A and 4B**), suggesting that DAF-12 may target the PMK-1 pathway to regulate the *C. elegans* immune response against *P. aeruginosa* infection. However, *daf-12* RNAi did not markedly change the *P. aeruginosa*-stimulated phosphorylation of PMK-1 (**Fig. 4C**), suggesting that DAF-12 might act upstream or parallel to PMK-1 to suppress the PMK-1/p38 MAPK pathway.

### MicroRNAs *let-7s* regulate both DAF-12 and NSY-1/PMK-1-mediated pathogenic defense

**Figure 5:**
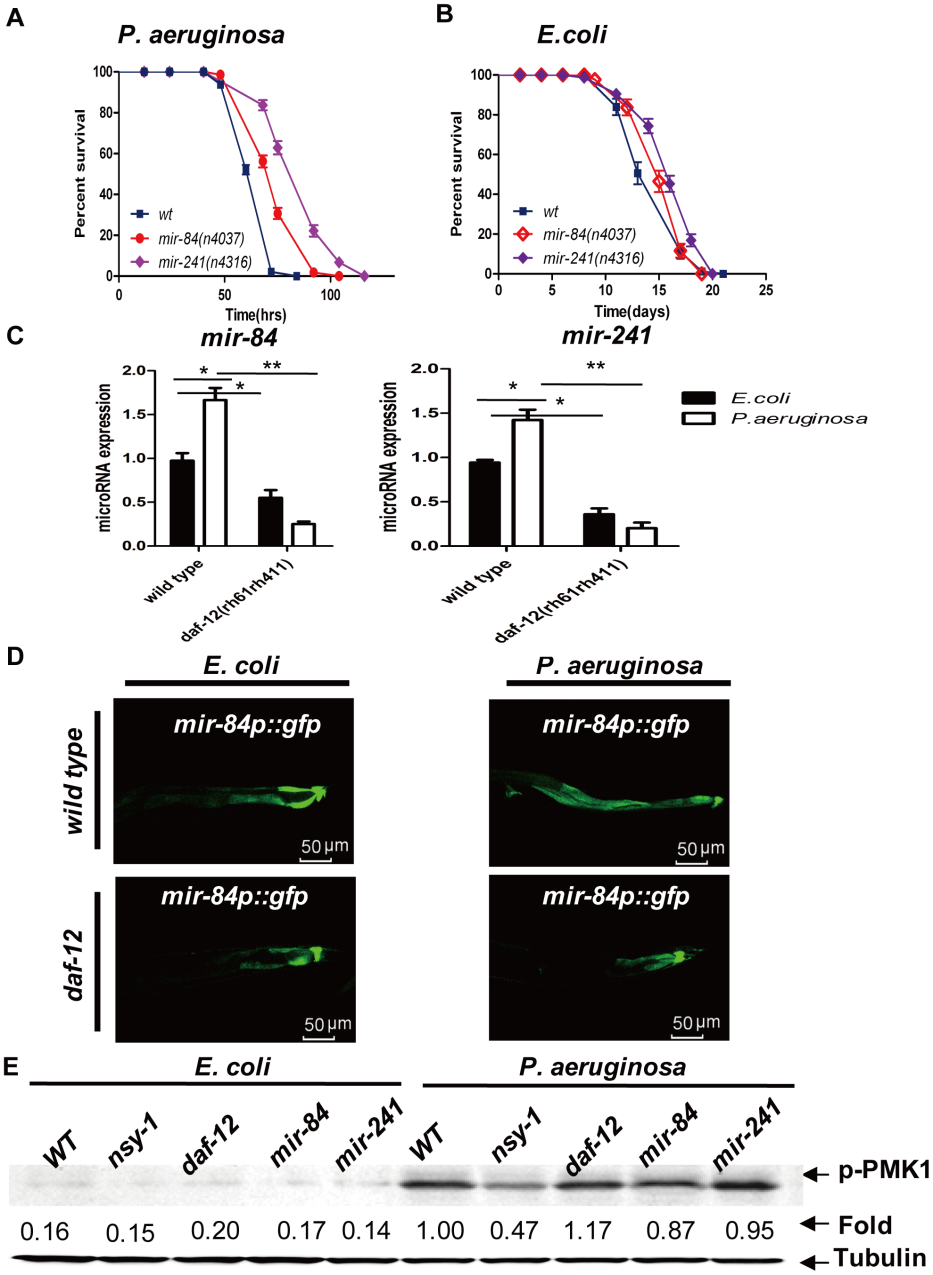
MicroRNAs *let-7s* regulate pathogenic defense. (**A**) Survival curve of N2, *mir-84*(*n4307*) (*P*<0.001) and *mir-241*(*n4316*) (*P*<0.001) on *P. aeruginosa*. (**B**) Lifespan assay of N2, *mir-84*(*n4037*) (*P*<0.001) and *mir-241*(*n4316*) (*P*<0.001) worms on *E. coli*. (**C**) qRT-PCR analysis of *mir-84* and *mir-241* expression in N2 and *daf-12*(*rh61rh411*) worms infected with *P. aeruginosa* for 24 hours. The data shown are the mean ± SEM of three independent experiments, each of which was performed in triplicate, *P<0.05 (**D**) Confocal microscopy of intestinal *mir-84p::GFP* expression in *daf-12(rh61rh411)* mutants on *E. coli* or *P. aeruginosa*. (**E**) Immunoblot analysis of the lysates from N2, *nsy-1*(RNAi), *daf-12*(*rh61rh411*), *mir-84*(*n4037*) and *mir-241*(*n4316*) worms on *E. coli* or *P. aeruginosa* using anti-phospho-p38 antibody and anti-tubulin antibody (loading control). All data shown are representative of at least three independent experiments (n≥50 adult nematodes per strain).

MicroRNAs are approximately 20- to 22-nucleotide-long RNA molecules that bind to the 3′ untranslated region (3′UTR) of target messenger RNAs (mRNAs) and that decrease their expression [34], [35]. DAF-12 activates the expression of the *let-7* miRNA homologs *mir-84* and *mir-241* (referred to as *let-7s*) to control developmental progression [29], [30]. To test whether *mir-84* or *mir-241* play a role in pathogenic defense, we infected the strains *mir-84(n4037)* and *mir-241(n4316)* with *P. aeruginosa*. Both *mir-84(n4037)* and *mir-241(n4316)* worms were more resistant to *P. aeruginosa* infection than the wild type (**Fig. 5A**). Likewise, both *mir-84(n4037)* and *mir-241(n4316)* worms had slightly longer lifespans than wild-type animals (**Fig. 5B**). We then employed the quantitative real-time PCR method to detect miRNA expression and found that *P. aeruginosa* infection of wild-type worms induced higher levels of *mir-84* and *mir-241* compared to *E. coli*. However, the *daf-12* mutation markedly reduced the expression of both *mir-84* and *mir-241* (**Fig. 5C**). Confocal microscopic imaging of *mir-84p::gfp* also indicated that the *mir-84* expression was highly upregulated in *P. aeruginosa*-infected wild-type worms, but not in the *daf-12(rh61rh411)* worms (**Fig. 5D** **, Fig. S5C**). To further determine the role of *let-7s* miRNAs in *C. elegans* innate immunity, we tested the function of *mir-48*, another *let-7* relative, in *P. aeruginosa* infection and found that the *mir-48(n4097)* mutant exhibited decreased resistance to *P. aeruginosa* (**Fig. S8A**), suggesting that the *let-7s* miRNAs may target different regulators of *C. elegans* innate immunity. Quantitative real-time PCR results showed that the expression of *daf-12*-tergeted antimicrobial genes was also upregulated in *let-7s* miRNAs mutants (**Fig. S9A**), suggesting that these genes are also targeted by *let-7s* miRNAs. We then fed the *daf-12* and *let-7s* mutants a GFP-tagged *P. aeruginosa* PA-14 strain and examined the bacterial burdens in worm intestines by confocal microscopy. We found there were significantly fewer accumulated bacteria in *daf-12(sa156)* and *mir-241(n4316)* worm intestines (**Fig. S10A and S10B**), suggesting that the inhibition of *daf-12* and *mir-241* may suppress bacterial accumulation through antimicrobial gene expression. The *nsy-1* RNAi also counteracted the pathogenic defense of *mir-84* mutant worms, and mutations of *mir-84* or *mir-241* did not affect the phosphorylation of PMK-1, suggesting that the *let-7s* miRNAs function downstream of DAF-12 to suppress the PMK-1/p38 MAPK signaling pathway (**Fig. 5E** **, Fig. S8B**).

### SKN-1 is a direct functional target of *let-7s*

**Figure 6:**
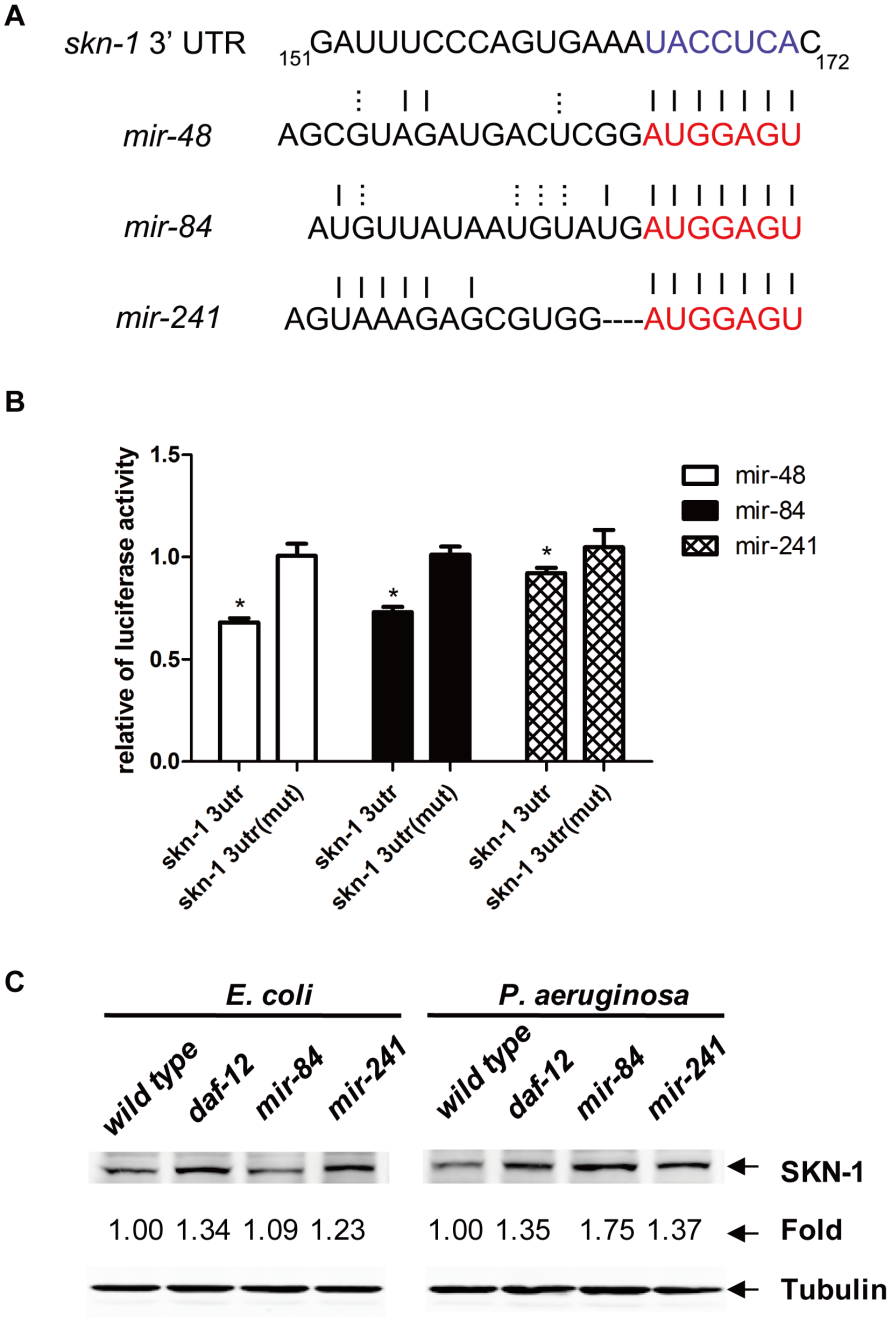
SKN-1 is a direct functional target of *let-7s* miRNAs. (**A**) Bioinformatics alignment of *let-7s* miRNAs and the 3′UTR of SKN-1. (**B**) Luciferase assays using the *skn-1* 3′UTR or the *skn-1* 3′UTR (mut) with *let-7s* mimics in HEK293T cells. The data shown are the mean ± SEM of three independent experiments, each of which was performed in triplicate, *P<0.05. (**C**) Immunoblot analysis of the lysates from N2, *daf-12*(RNAi), *mir-84*(*n4037*) and *mir-241*(*n4316*) worms on *E. coli* or *P. aeruginosa* using anti-SKN-1 antibody and anti-tubulin antibody (loading control).

The finger protein *hbl-1* is one target of miRNAs *let-7s*, and the expression of *hbl-1* is regulated by *daf-12* and *let-7s* [29]. The inhibition of *hbl-1* by RNAi reduced the pathogenic resistance, but not the lifespan of *C. elegans* (**Fig. S11A and S11B**). To identify other target genes of *let-7* family of miRNAs, we performed a bioinformatics analysis, determining that *skn-1* is a potential target (**Fig. 6A**). To determine whether *let-7s* miRNAs could bind to the 3′-UTR of the *skn-1* mRNA and suppress it, we fused the 3′-UTR region of the *C. elegans skn-1* mRNA to the 3′-end of a luciferase reporter gene and co-transfected it with synthesized dsRNAs mimicking *let-7s* miRNAs (*let-7*s mimics) into HEK293T cells. In contrast to the luciferase activity in the 3′-UTR seed region mutants (*skn-1* 3′-UTR (mut)), which could not bind with and respond to *let-7s* miRNAs, the luciferase activity of the *skn-1* 3′-UTR decreased by approximately 30% in response to *mir-48* mimics or *mir-84* mimics and by approximately 10% in response to *mir-241* mimics (**Fig. 6B**). Western blot results also showed that the SKN-1 protein expression could be upregulated by inhibition of *daf-12*, *mir-84* and *mir-241* (**Fig. 6C** **, Fig. S11C**). These results suggested that *skn-1* is a target of *mir-84* and *mir-241*.

### DAF-12 regulates pathogenic resistance through SKN-1

**Figure 7:**
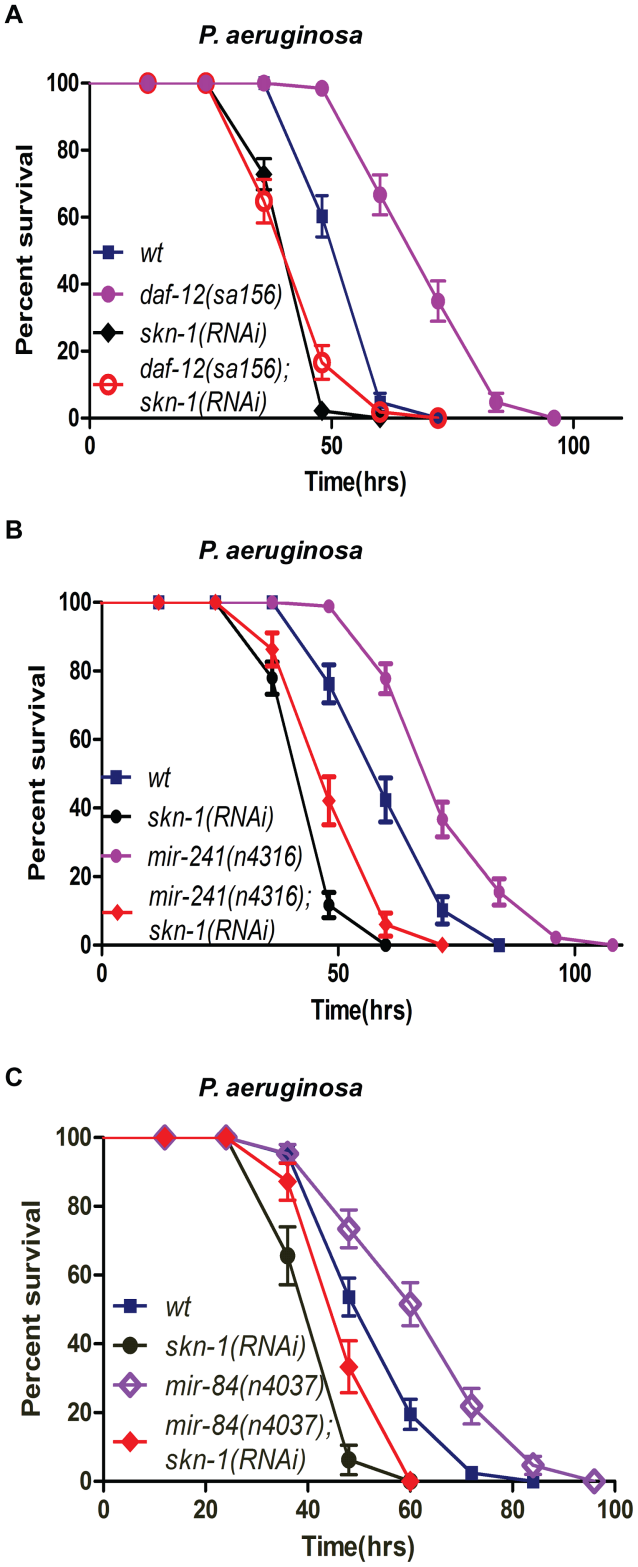
DAF-12 regulates pathogenic resistance through SKN-1. (**A**) Survival curve of N2, *daf-12*(*sa156*) (*P*<0.001), *skn-1*(RNAi) (*P*<0.001) and *daf-12*(*sa156*); *skn-1*(RNAi) worms (*P* = 0.745 compared to *skn-1* (RNAi)) on *P. aeruginosa*. (**B**) Survival curve of wild-type N2, *mir-241*(*n4315*) (*P*<0.001), *skn-1*(RNAi) (*P*<0.001) and *mir-241*(*n4315*); *skn-1(RNAi)* worms (*P* = 0.008 compared to *skn-1* RNAi) on *P. aeruginosa*. (**C**) Survival curve of wild-type N2, *mir-84*(*n4037*) (*P*<0.001), *skn-1*(RNAi) (*P*<0.001) and *mir-84*(*n4037*); *skn-1(RNAi)* worms (*P*<0.0001 compared to *skn-1* RNAi) on *P. aeruginosa*. All data shown are representative of at least three independent experiments (n≥50 adult nematodes per strain).

SKN-1 is a kinase substrate of PMK-1 and regulates *C. elegans* resistance to oxidative stress [36], [37]. The inhibition of *skn-1* by RNAi markedly attenuated the pathogenic resistance of *C. elegans* (**Fig. S12A**) [38] but did not affect the pathogenic resistance of *pmk-1(km25)* mutants (**Fig. S12B**). In worms infected with *P. aeruginosa* but not *E. coli*, SKN-1 accumulated in the nuclei of intestinal cells (**Fig. S12C and S12D**) [38], [39]. However, *nsy-1* RNAi attenuated the nuclear accumulation of SKN-1 (**Fig. S12E**), suggesting that *skn-1* may also act downstream of NSY-1/PMK-1 to regulate the immune response. Conversely, *skn-1* RNAi markedly reversed the enhanced pathogenic resistance of the *daf-12(sa156)* mutant as well as that of the *mir-84* or *mir-241* mutants (**Fig. 7A, 7B and 7C**). Quantitative real-time RT-PCR results showed that the inhibition of *daf-12* and of *let-7s* miRNAs significantly increase the expression of *gcs-1*, a SKN-1 downstream gene (**Fig. S13A**), suggesting that the DAF-12 and *let-7s* miRNAs may suppress SKN-1 activity. We treated the *skn-1::gfp* transgenic worms [37] with *daf-12* RNAi and confocal imaging analysis revealed that *daf-12* RNAi treatment dramatically increased both the expression and nuclear accumulation of SKN-1 (**Fig. S13B and S13C**). These findings suggest that DAF-12-*let-7s* may target SKN-1, thus counteracting the activation of SKN-1 by the NSY-1/PMK-1 pathway.

## Discussion

We have identified DAF-12 as a novel negative regulator of innate immune signaling pathways in *C. elegans*. DAF-12, along with NHR-8 and NHR-48, is a conserved homolog of the mammalian vitamin D/liver X receptor (LXR) in *C. elegans* [6], [7]. The functions of vitamin D and LXR in mammalian innate immunity have been extensively investigated [40], [41]. In a variety of human innate immune cell types (i.e., macrophages and monocytes), vitamin D stimulates antibacterial activity by increasing the expression of antimicrobial genes and by promoting autophagic mechanisms. Furthermore, vitamin D insufficiency, which is a global health issue, may increase the risk of many infectious diseases [40]. Whereas *daf-12* negatively regulates the innate immunity of *C. elegans*, a mutation of either of *nhr-8* or *nhr-48* impairs the *C. elegans* host defense (**Fig. S2A**), suggesting that the regulatory role of nuclear hormone receptors may depend on their ligands and their target genes.

Hormone binding to nuclear hormone receptors regulates the *C. elegans* reproductive life cycle and entry into dauer diapauses [42]. We have shown that the sterol-derived dafachronic acids (DAs), the ligand of DAF-12, negatively regulate the pathogenic resistance of *C. elegans* in a DAF-12-dependent manner. In response to pathogenic infection, DAF-12 translocated from the nucleus into the cytoplasm. Although the regulation of DAF-12 translocation is not fully understood, the binding of the DAs and their co-factors is hypothesized to lead to the translocation of DAF-12. DAF-9, which synthesizes DAs, has also been shown to regulate *C. elegans* antibacterial activity, suggesting that the sterol hormones might be the key sensors of pathogenic infection and important regulators of pathogenic defense.

DAF-12 is known to activate *let-7s* miRNAs and thus regulate the developmental progression through downstream target *hlb-1* [29], [30]. In this study, we showed that a *P. aeruginosa* infection induces the intestinal expression of *mir-84* and *mir-241*, which is consistent with Kudlow et al.'s earlier findings that multiple miRNAs accumulate in the intestinal miRISCs upon infection [43] and that DAF-12-mediated immunity is dependent on the activation of its downstream miRNA *let-7s*. Although our understanding of the role of miRNAs in the molecular signaling pathways of the immune response is rapidly expanding [24], to the best of our knowledge, this is the first evidence of the involvement of miRNAs in the innate immune regulation in *C. elegans*. We have also observed that more DAF-12 accumulate in the nuclei of neurons or intestinal cells when worms were fed *E. coli* but in a diffuse distribution in *P. aeruginosa*-infected worms. However, there may be still enough DAF-12 present in the nuclei of the intestine cell, which would be responsible for the induction of *let-7s* miRNAs.

Furthermore, we have demonstrated that SKN-1 is a direct target of *let-7s* miRNAs. SKN-1 accumulate in the nuclei of intestinal cells of worms infected with *P. aeruginosa*, but not *E. coli*, which is consistent with Papp et al. and Haeven et al.'s reports that exposure to *P. aeruginosa* leads to SKN-1 accumulation in intestinal nuclei [38], [39]. Their data have also shown that PA14 infection triggers the transcriptional activation of *gcs-1* and *gst-4*, two downstream target gene of SKN-1. However, data in our experiments suggested that infection of *P. aeruginosa* may induce more *let-7s* miRNAs and thus downregulate the production of SKN-1. One possible explanation is that even if the SKN-1 production is downregulated during the infection, the activity of SKN-1 is more dependent on its protein modification rather than its quantity. Regulation of SKN-1 at both the level of its activity and quantity precisely modulate the innate immune response to microbial infection.. Furthermore, we found that the inhibition of *skn-1* by RNAi markedly reduced DAF-12/*let-7s*-mediated pathogenic defense. These findings provide evidence that nuclear hormone receptors control *let-7s* miRNAs regulation of the *C. elegans* innate immunity, suggesting that DAF-12 may couple developmental progression and the response to pathogenic infection in order to coordinate appropriate immune responses.

The oxidative stress response is an evolutionally conserved response to reactive oxygen species (ROS), which are produced by mitochondrial respiration, toxins and pathogen virulence factors [44]. SKN-1 is required for the proper response of *C. elegans* to oxidative stress, which is mediated by the NSY-1/PMK-1 and DAF-2 insulin-like signaling pathways [36], [37]. Our present findings demonstrate an essential role of SKN-1 in the pathogenic resistance of *C. elegans*, an observation that is consistent with two other independent studies [38], [39]. Thus, SKN-1 appears to integrate longevity, stress resistance and pathogenic resistance. Although the molecular pathway by which SKN-1 regulates the innate response to pathogens remains unclear, an SKN-1-mediated oxidative stress response could potentially protect the worms from the peroxidation damage caused by ROS during pathogenic infection. Further investigation of the common downstream target of various SKN-1 actions is required to elucidate the role of SKN-1 in the pathogenic resistance of *C. elegans*.

In summary, our data demonstrate that DAF-12 and its steroidal ligands, DAs, negatively regulate the innate immune responses of *C. elegans* to pathogenic infection. DAF-12 appears to activate *let-7s* miRNAs to directly target SKN-1, a component of the NSY-1/PMK-1 immune signaling pathway, thus regulating the pathogenic resistance of *C. elegans* (**Fig. S14**). These findings not only reveal a novel signaling pathway in the *C. elegans* defense against pathogens but also provide a link between endocrine signaling and innate immune responses, thus integrating developmental progression and pathogenic resistance.

## Materials and Methods

### Materials

(25S)- Δ^4^- and Δ^7^-DAs were produced in the Knölker laboratory [45]. All *C. elegans* strains were obtained from Caenorhabditis Genetics Center (CGC) unless otherwise noted.

### Nematode methods

The *C. elegans* strains used in this study are listed in Table S2. All of the strains were maintained at 20°C using standard methods unless otherwise noted.

### Lifespan and *P. aeruginosa* killing assay

Lifespan and *P. aeruginosa* killing assays were conducted at least three times, as previously described [46]. A *P* value less than or equal to 0.05 was considered statistically significant. Statistical analysis of lifespan and *P. aeruginosa* killing assay is shown in Table S3, S4, S6 and S7.

### Screening of a transcription factor RNAi library

RNAi of candidate transcription factors in N2 worms was carried out using standard bacterial feeding methods. For all feeding assays, worms were exposed to RNAi bacteria from the time of hatching. Synchronized young adult animals were transferred to *P. aeruginosa* lawns supplemented with 50 µg/ml 5-fluorodeoxyuridine (FUDR, Sigma). *P. aeruginosa* killing assays were performed as described above.

### Confocal microscopy assay

Worms were washed from their plates with M9, anaesthetized with M9 containing 0.1% NaN_3_, fixed in the 2% soft agar and subjected to confocal imaging assay. Images were captured using Leica TCS SP5.

### Transmission electron microscopy assay

Wild-type N2 and *daf-12(sa156)* young adults were fed *P. aeruginosa* or *E. coli* for 48 hours. Worms were rinsed from plates with M9 buffer, and anaesthetized in 8% alcohol in M9. Fixation and Sectioning was performed with a conventional two-steps method as described in Worm Method. Photographs were captured using HITACHI H-7650.

### Dafachronic acids assay

Wild-type N2 and *daf-12(sa156)* young adults were removed from plates and bathed with Δ4-DAs, Δ7-DAs or cholesterol (400 nM) in M9 8 hours before killing assay. The killing assay was performed on *P. aeruginosa* plates supplemented with Δ^4^-DAs, Δ^7^-DAs or cholesterol (400 nM).

### Luciferase assay

A 0.5-kb region of the *skn-1* 3′ UTR containing the predicted miRNA *let-7s* binding sites was cloned into the psi-CHECK2 to obtain the *skn-1* 3′ UTR-luc construct. The *skn-1* 3′ UTR (mut)-luc construct was obtained from *skn-1* 3′UTR construct by mutating the complementary sequence of *let-7s*' seed region(TACCTCA to TAGGTGA). Constructs were co-transfected with synthesized dsRNAs mimicking the *let-7s* miRNAs to HEK293T cells and the luciferase assay was performed using the dual-luciferase reporter assay system (Promega).

### Quantitative real-time PCR of antimicrobial peptide expression

Synchronized *C. elegans* animals were treated essentially as described above for the killing assays except for the omission of FUDR. Infected samples were compared to control samples fed on the same medium with *E. coli* OP50-1. Total RNA was extracted as described [31] and reverse transcribed using the ReverTra Ace Q-PCR RT kit (Toyobo). cDNA was subjected to qRT-PCR analysis as described [31]. The primer sequences are listed in Table S5. All values were normalized to *act-1*. One-tailed *t*-tests were performed with GraphPad Prism4. A *P* value less than or equal to 0.05 was considered significant.

### Quantitative real-time PCR for microRNA *let-7s*

Synchronized worms were collected in TRIzol (Invitrogen) and treated as described [47]. The miRNeasy Mini kit (QIAGEN) and TaqMan MicroRNA Reverse Transcription kit (Applied Biosystems) were used for total RNA and cDNA preparation, respectively. qRT-PCR was performed with Power SYBR Green master mix (Applied Biosystems) on a 7900HT Fast Real-Time PCR System (Applied Biosystems). Sno-RNA U18 was used as an internal control. The primer sequences were gifted from Prof. Adam Antebi from the Max Planck Institute for Biology of Ageing. One-tailed *t*-tests were performed with GraphPad Prism4. A *P* value less than or equal to 0.05 was considered significant.

### Western blot analysis of PMK-1 activation and SKN-1 expression

Synchronized L4 populations of wild-type N2, *daf-12*(*RNAi*), *daf-12*(*rh61rh411*), *mir-84*(*n4037*) and *mir-241*(*n4316*) animals were infected with *P. aeruginosa* as described [1]. Western blot analyses of activated p38 MAPK were performed as described [46]. Western blot analyses of SKN-1 expression were performed using anti-SKN-1 (Santa Cruz).

## Supporting Information

Figure S1 **RNAi treatment of DAF-12 increased** ***C. elegans*** **lifespan.** (**A**) Lifespan of N2 worms treated with *daf-12* RNAi (*P*<0.0001) and control vector. (**B**) Survival curve of wild-type N2 and *daf-12(dhIs26)* (*P*<0.0001) worms on *P. aeruginosa*.
(TIF)

Figure S2 **NHR-8 and NHR-48 regulate** ***C. elegans*** **innate immunity.** (**A**) Survival curve of wild-type N2, *nhr-8(ok186)* (*P*<0.0001) and *nhr-48(ok178)* (*P*<0.0001) worms on *P. aeruginosa*. (**B**) Survival curve of wild-type N2 and *daf-12(sa156)* (*P*<0.0001) worms on *S. aureus*.
(TIF)

Figure S3 **DAF-12 mutations have different roles in regulation of aging process and innate immunity.** (**A**) Survival curve of wild-type N2, *daf-12(m20)* (*P*<0.0001), *daf-12(m25)* (*P*<0.0001) and *daf-12(sa204)* (*P*<0.0001) mutants on *E. coli*. (**B**) Survival curve of wild-type N2, *daf-12(m20)* (*P* = 0.4783), *daf-12(m25)* (*P* = 0.2637) and *daf-12(sa204)* (*P*<0.001) mutants on *P. aeruginosa*.
(TIF)

Figure S4 ***P. aeruginosa*** **infection induces DAF-12 translocation.** (**A**) Confocal imaging of DAF-12::gfp transgenic worm fed *E. coli* or *P. aeruginosa* for 24 hours. (**B**) Quantification of *daf-12::gfp* translocation observed in worms fed *E. coli* (n = 9) or *P. aeruginosa* (n = 13) in **Fig. S4A**. (**C**) Western blot assay of daf-12-GFP of DAF-12::gfp transgenic worm fed *E. coli* or *P. aeruginosa* for 24 hours using anti-GFP antibody.
(TIF)

Figure S5 **Quantification of GFP signals.** (**A**) Quantification of F55G11.7::GFP signals in **Fig. 2B**. (**B**) Quantification of DOD-22::GFP signals in **Fig. 2B**. (**C**) Quantification of *mir-84p*::GFP signals in **Fig. 5D**.
(TIF)

Figure S6 **An increased dose of Δ^7^-DA does not lead to further increases in pathogenic susceptibility.** Survival curve of wild-type N2 worm with cholesterol (400 nM), Δ^7^-DAs (400 nM) (*P* = 0.007) and Δ^7^-DAs (1 µM) (*P* = 0.5998 compared to 400 nM) on *P. aeruginosa*.
(TIF)

Figure S7 **DAF-16 RNAi has no effect on increased resistance to** ***P. aeruginosa*** **of DAF-12 mutants.** (**A**) *P. aeruginosa* killing assay of N2, *din-1*(*dh127*) (*P* = 0.0823), *daf-12(RNAi)* (*P*<0.0001) and *din-1(dh127)*;*daf-12(RNAi)* (*P* = 0.002 compared to *daf-12* RNAi) animals. (**B**)Survival curve of wild-type N2, *daf-16*(RNAi) (*P* = 0.0485), *daf-12(sa156)* (*P*<0.0001) and *daf-12(sa156)*;*daf-16*(RNAi) (*P* = 0.9012 compared to *daf-12(sa156)*) worms on *P. aeruginosa*.
(TIF)

Figure S8 **Mir-84 regulates innate immunity through PMK-1 pathway.** (**A**) Survival curve of wild-type N2 and *mir-48(n4097)* (*P*<0.0001) worms on *P. aeruginosa*. (**B**) Survival curve of wild-type N2, *nsy-1*(RNAi) (*P*<0.001), *mir-84(n4037)* (*P*<0.0001) and *mir-84(n4037)*;*nsy-1*(RNAi) (*P* = 0.1114 compared to *mir-84(n4037)*) upon *P. aeruginosa* infection.
(TIF)

Figure S9 **MiRNAs** ***let-7s*** **and SKN-1 regulate DAF-12-mediated AMPs expression.** (**A**) Quantitative real-time PCR assay of antimicrobial gene expression of wild-type N2, *mir-84(n4037)* and *mir-241(n4316)* young adults fed *E. coli* or *P. aeruginosa* for 24 hours. (**B**) Quantitative real-time PCR assay of antimicrobial gene expression of wild-type N2 and *skn-1*(RNAi) young adults fed *E. coli* or *P. aeruginosa* for 24 hours.
(TIF)

Figure S10 **DAF-12 and** ***let-7s*** **miRNAs regulate bacterial accumulation in worm intestine.** (**A**) Confocal imaging of wild-type N2, *daf-12(sa156)*, *mir-84(n4037)* and *mir-241(n4316)* animals fed GFP-tagged *P. aeruginosa* for 24 hours. (**B**) Quantification of GFP signals in Supp. Fig. 10A.
(TIF)

Figure S11 **HBL-1 regulates innate immunity.** (**A**) Survival curve of N2 and *hbl-1*(RNAi) (*P*<0.0001) worms on *P. aeruginosa*. (**B**) Lifespan assay of N2 and *hbl-1*(RNAi) (*P* = 0.8882) worms on *E. coli*. (**C**) Immunoblot analysis of the lysates from N2, *skn-1*(RNAi) and *daf-12(rh61rh411)* young adults fed *E. coli* or *P. aeruginosa* using anti-SKN-1 antibody and anti-tubulin antibody (loading control).
(TIF)

Figure S12 **SKN-1 acts at downstream of PMK-1 to regulate innate immunity.** (**A**) Survival curve of N2 and *skn-1*(RNAi) (*P*<0.0001) worms on *P. aeruginosa*. (**B**) Survival curve of wild-type N2, *pmk-1*(*km25*) (*P*<0.0001) and *pmk-1*(*km25*);*skn-1*(RNAi) (*P* = 0.0067) worms on *P. aeruginosa*. (**C–E**) Confocal microscopy of *nsy-1* RNAi-treated or control RNAi-treated *skn-1::GFP* worms on *P. aeruginosa* or *E. coli*. Arrows shows the nuclear *skn-1*::*GFP*.
(TIF)

Figure S13 **DAF-12 regulates SKN-1 activity.** (**A**) Quantitative real-time PCR assay of *gcs-1* expression of wild-type N2, *daf-12(RNAi)*, *mir-84(n4037)* and *mir-241(n4316)* young adults fed *E. coli* or *P. aeruginosa* for 24 hours. (**B**) Confocal imaging of *daf-12* RNAi treated or control treated young adults of *skn-1::gfp* transgenic worms. Arrows shows the nuclear *skn-1*::*GFP*. (**C**) Quantification of *skn-1::gfp* observed in worms treated with *daf-12* RNAi (n = 23) or control (n = 19) in **Fig. S13B**.
(TIF)

Figure S14 **The hypothesized diagram.** *Caenorhabditis elegans* nuclear receptor DAF-12 negatively regulates the pathogenic defense via its downstream microRNAs, *let-7*s, which may directly target SKN-1, thus counteract the activation of SKN-1 by NSY-1/PMK-1 pathway.
(TIF)

Table S1 **List of alleles isolated from the RNAi screening.**
(TIF)

Table S2 **List of all the** ***C. elegans*** **strains used in this study.**
(TIF)

Table S3 **The statistical analysis of all** ***P. aeruginosa*** **killing assays shown in figures.**
(TIF)

Table S4 **The statistical analysis of all lifespan assays shown in figures.**
(TIF)

Table S5 **The combination of primers used in quantitative real-time RT-PCR assay.**
(TIF)

Table S6 **The statistical analysis of all** ***P. aeruginosa*** **killing assays shown in supplementary figures.**
(TIF)

Table S7 **The statistical analysis of all lifespan assays shown in supplementary figures.**
(TIF)
