# Supplementary figures and images for: Nuclear Hormone Receptor Regulation of MicroRNAs Controls Innate Immune Responses in C. elegans
Source: PLoS Pathog. 2013 Aug 22;9(8):e1003545. doi: 10.1371/journal.ppat.1003545 (PMC3749966; doi:10.1371/journal.ppat.1003545)

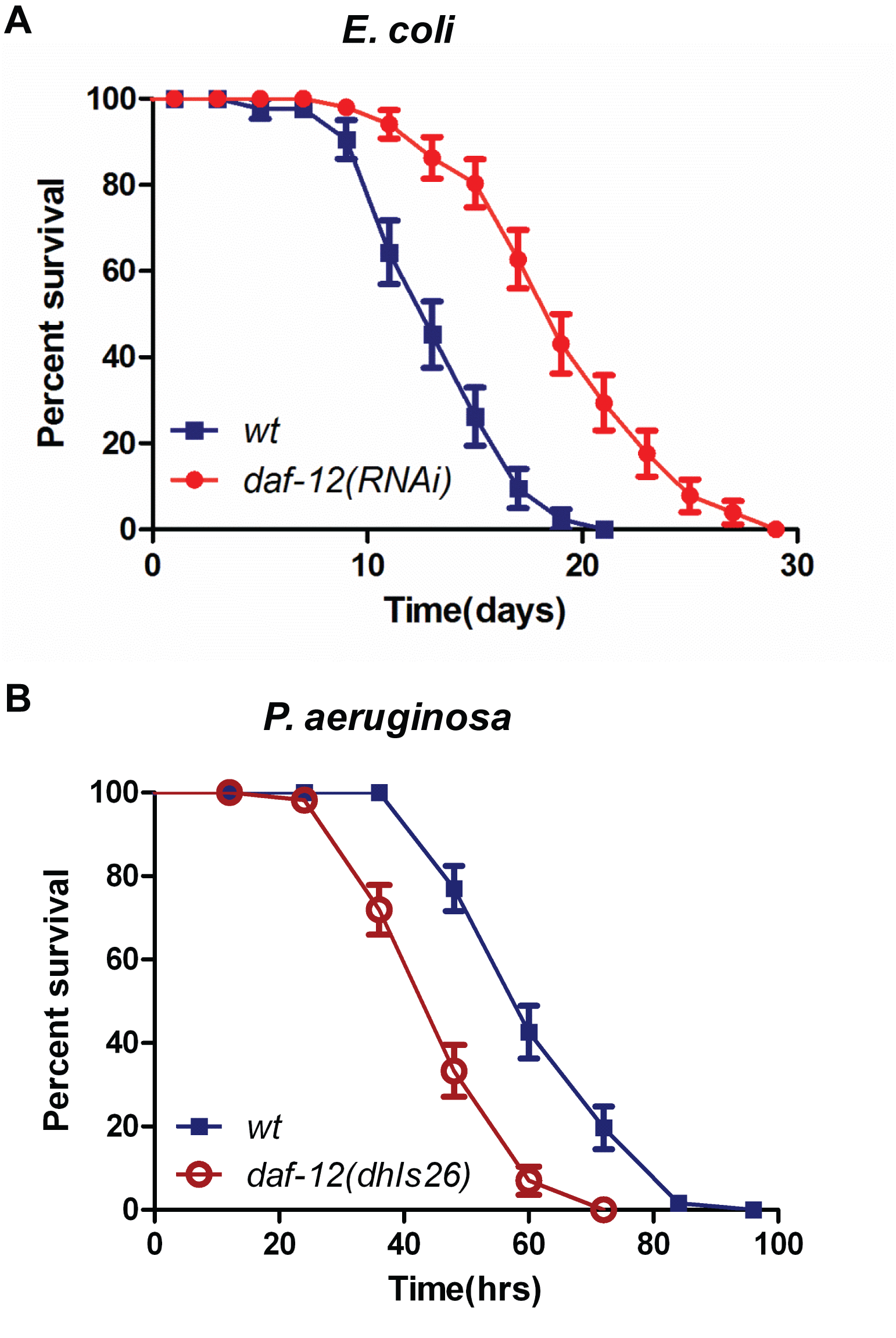

Supplement: Figure S1 — RNAi treatment of DAF-12 increased C. elegans lifespan. (A) Lifespan of N2 worms treated with daf-12 RNAi (P<0.0001) and control vector. (B) Survival curve of wild-type N2 and daf-12(dhIs26) (P<0.0001) worms on P. aeruginosa. (TIF) [file ppat.1003545.s001.tif]

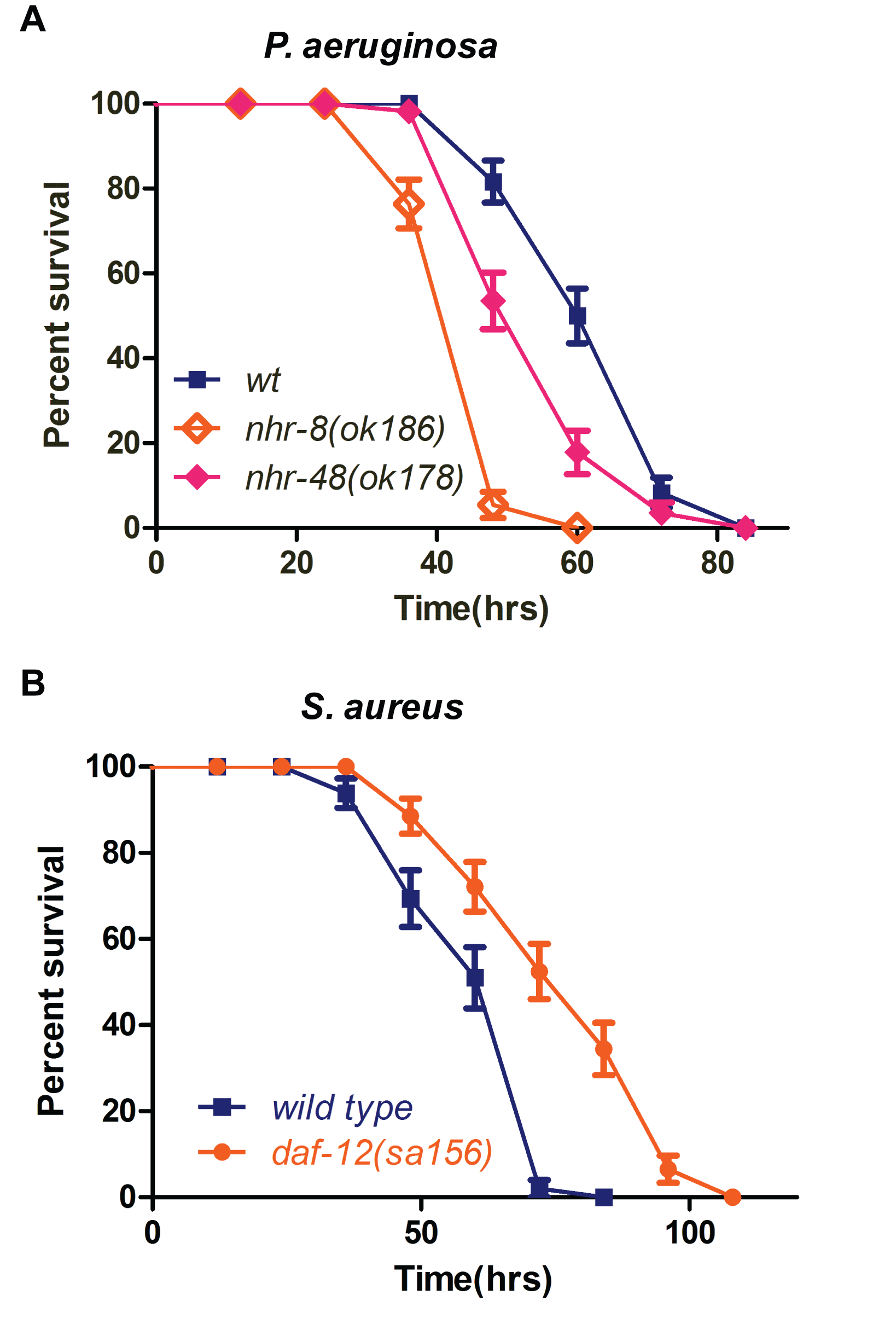

Supplement: Figure S2 — NHR-8 and NHR-48 regulate C. elegans innate immunity. (A) Survival curve of wild-type N2, nhr-8(ok186) (P<0.0001) and nhr-48(ok178) (P<0.0001) worms on P. aeruginosa. (B) Survival curve of wild-type N2 and daf-12(sa156) (P<0.0001) worms on S. aureus. (TIF) [file ppat.1003545.s002.tif]

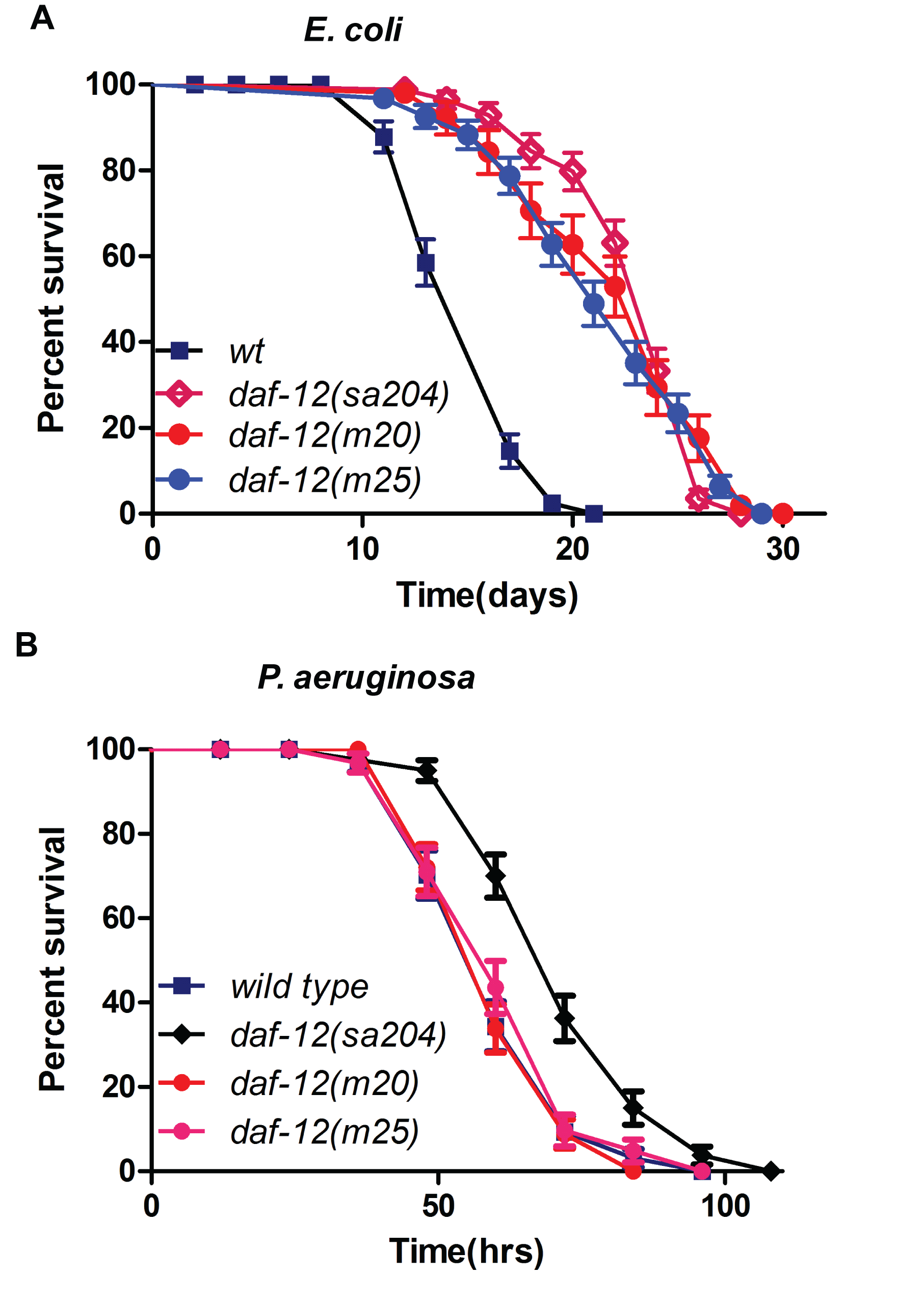

Supplement: Figure S3 — DAF-12 mutations have different roles in regulation of aging process and innate immunity. (A) Survival curve of wild-type N2, daf-12(m20) (P<0.0001), daf-12(m25) (P<0.0001) and daf-12(sa204) (P<0.0001) mutants on E. coli. (B) Survival curve of wild-type N2, daf-12(m20) (P = 0.4783), daf-12(m25) (P = 0.2637) and daf-12(sa204) (P<0.001) mutants on P. aeruginosa. (TIF) [file ppat.1003545.s003.tif]

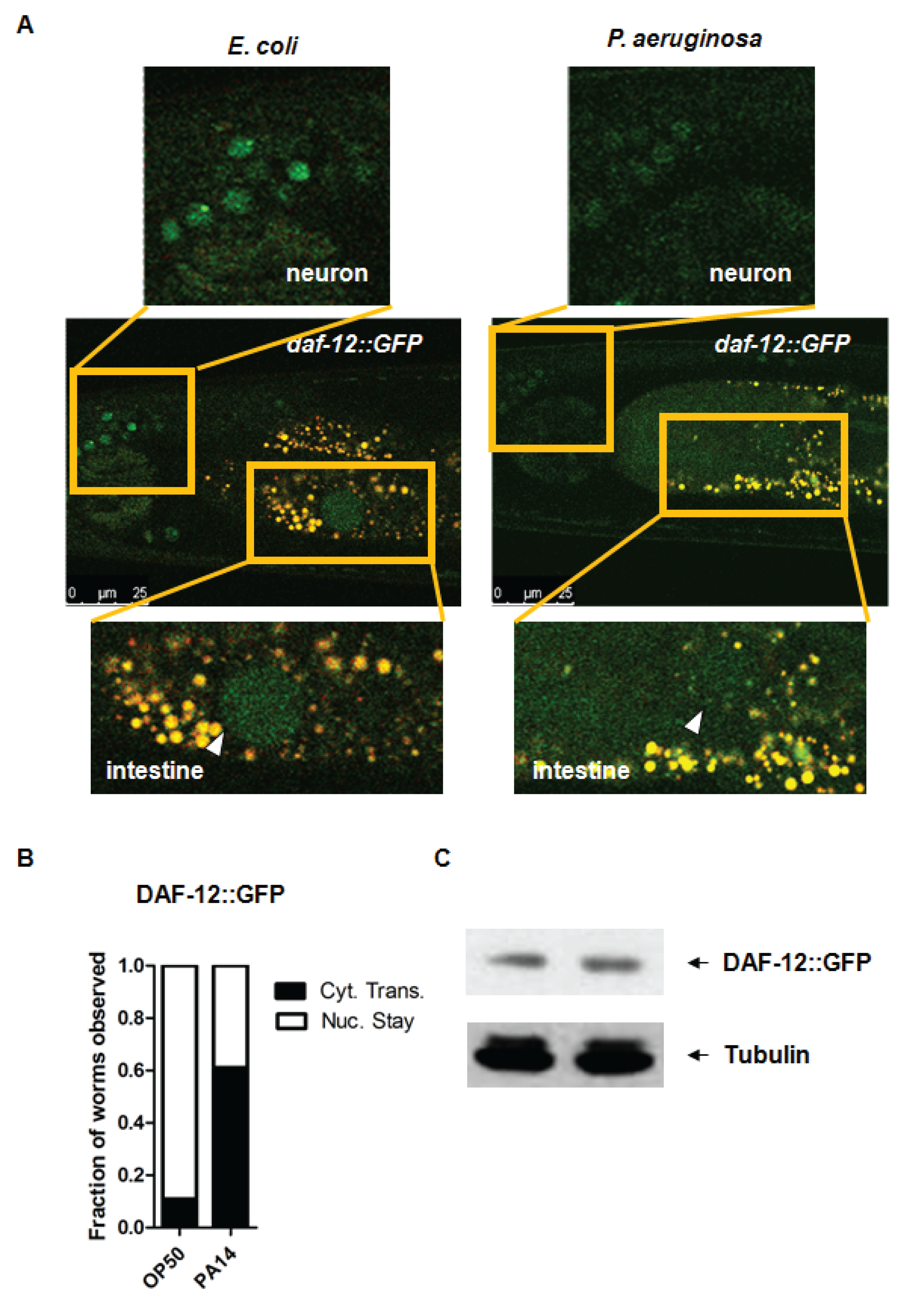

Supplement: Figure S4 — P. aeruginosa infection induces DAF-12 translocation. (A) Confocal imaging of DAF-12::gfp transgenic worm fed E. coli or P. aeruginosa for 24 hours. (B) Quantification of daf-12::gfp translocation observed in worms fed E. coli (n = 9) or P. aeruginosa (n = 13) in Fig. S4A. (C) Western blot assay of daf-12-GFP of DAF-12::gfp transgenic worm fed E. coli or P. aeruginosa for 24 hours using anti-GFP antibody. (TIF) [file ppat.1003545.s004.tif]

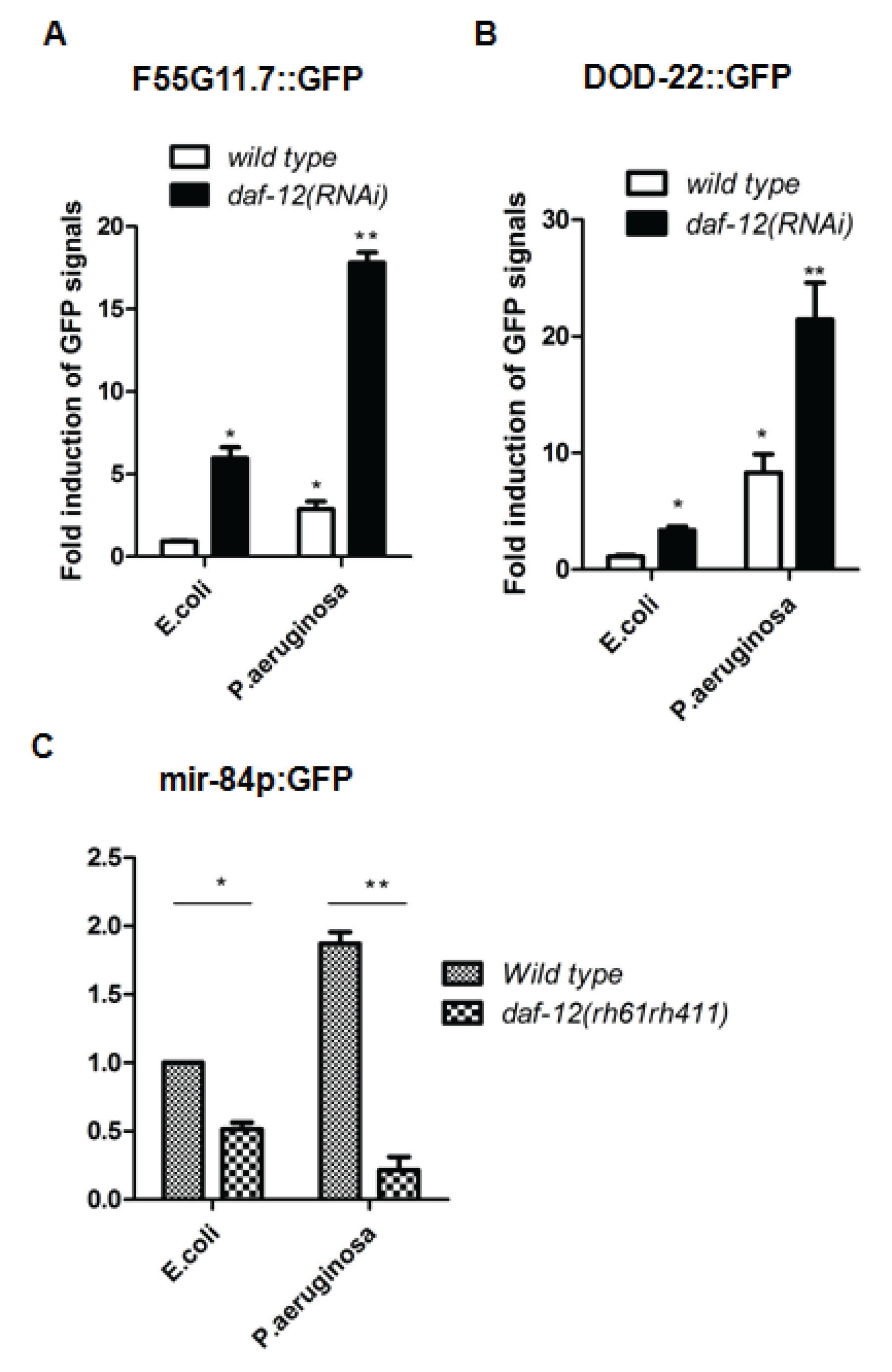

Supplement: Figure S5 — Quantification of GFP signals. (A) Quantification of F55G11.7::GFP signals in Fig. 2B . (B) Quantification of DOD-22::GFP signals in Fig. 2B . (C) Quantification of mir-84p::GFP signals in Fig. 5D . (TIF) [file ppat.1003545.s005.tif]

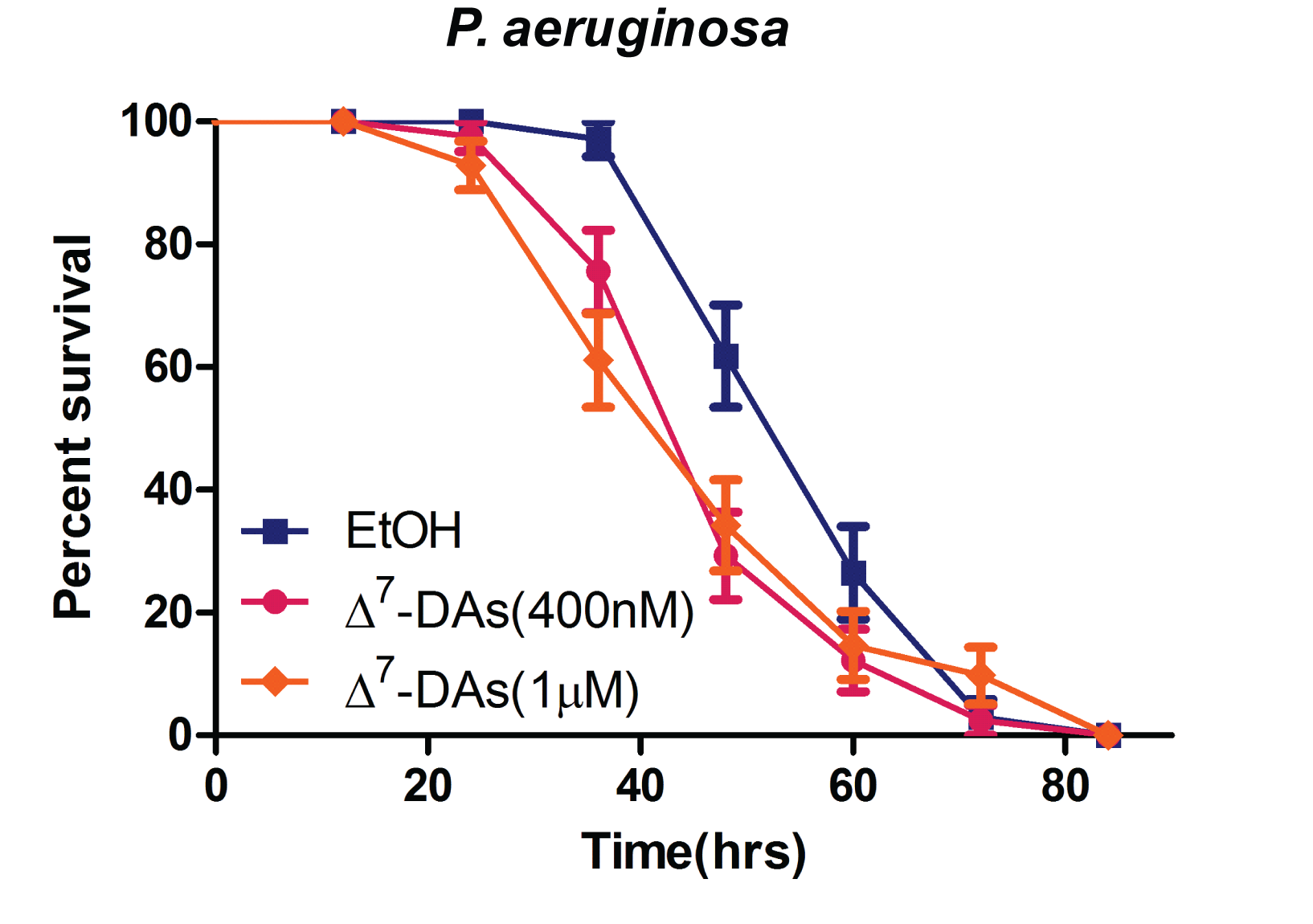

Supplement: Figure S6 — An increased dose of Δ7-DA does not lead to further increases in pathogenic susceptibility. Survival curve of wild-type N2 worm with cholesterol (400 nM), Δ7-DAs (400 nM) (P = 0.007) and Δ7-DAs (1 µM) (P = 0.5998 compared to 400 nM) on P. aeruginosa. (TIF) [file ppat.1003545.s006.tif]

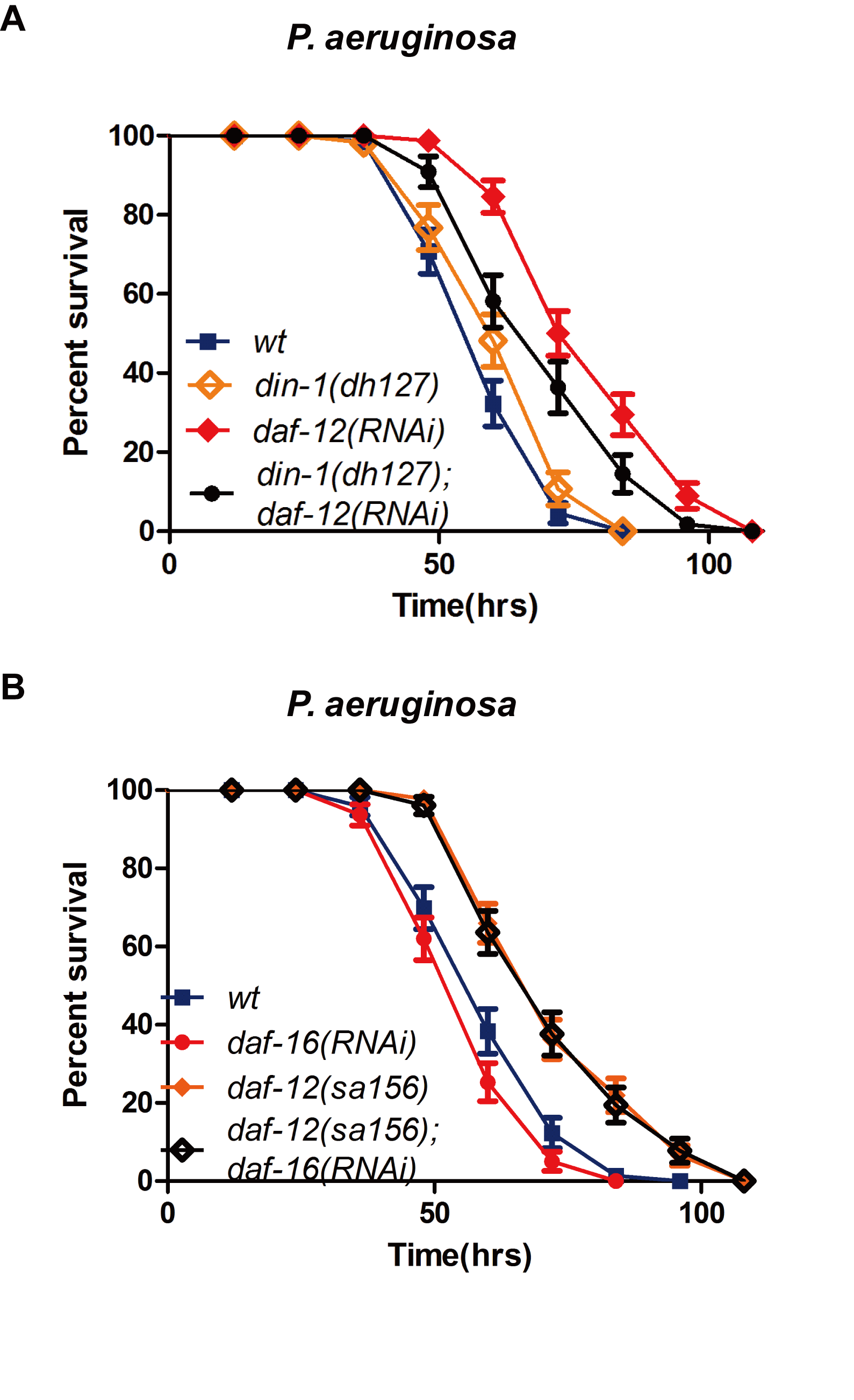

Supplement: Figure S7 — DAF-16 RNAi has no effect on increased resistance to P. aeruginosa of DAF-12 mutants. (A) P. aeruginosa killing assay of N2, din-1(dh127) (P = 0.0823), daf-12(RNAi) (P<0.0001) and din-1(dh127);daf-12(RNAi) (P = 0.002 compared to daf-12 RNAi) animals. (B)Survival curve of wild-type N2, daf-16(RNAi) (P = 0.0485), daf-12(sa156) (P<0.0001) and daf-12(sa156);daf-16(RNAi) (P = 0.9012 compared to daf-12(sa156)) worms on P. aeruginosa. (TIF) [file ppat.1003545.s007.tif]

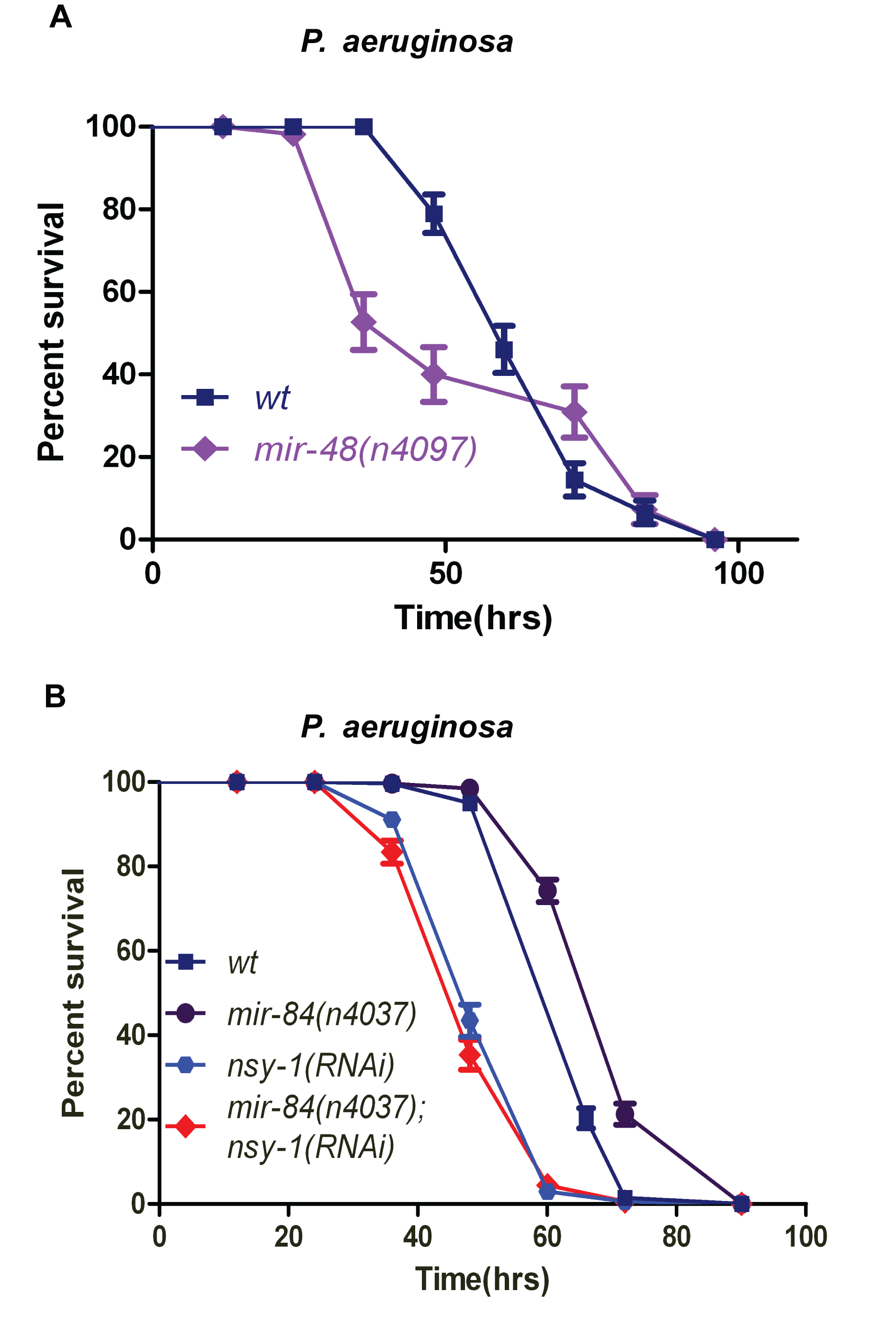

Supplement: Figure S8 — Mir-84 regulates innate immunity through PMK-1 pathway. (A) Survival curve of wild-type N2 and mir-48(n4097) (P<0.0001) worms on P. aeruginosa. (B) Survival curve of wild-type N2, nsy-1(RNAi) (P<0.001), mir-84(n4037) (P<0.0001) and mir-84(n4037);nsy-1(RNAi) (P = 0.1114 compared to mir-84(n4037)) upon P. aeruginosa infection. (TIF) [file ppat.1003545.s008.tif]

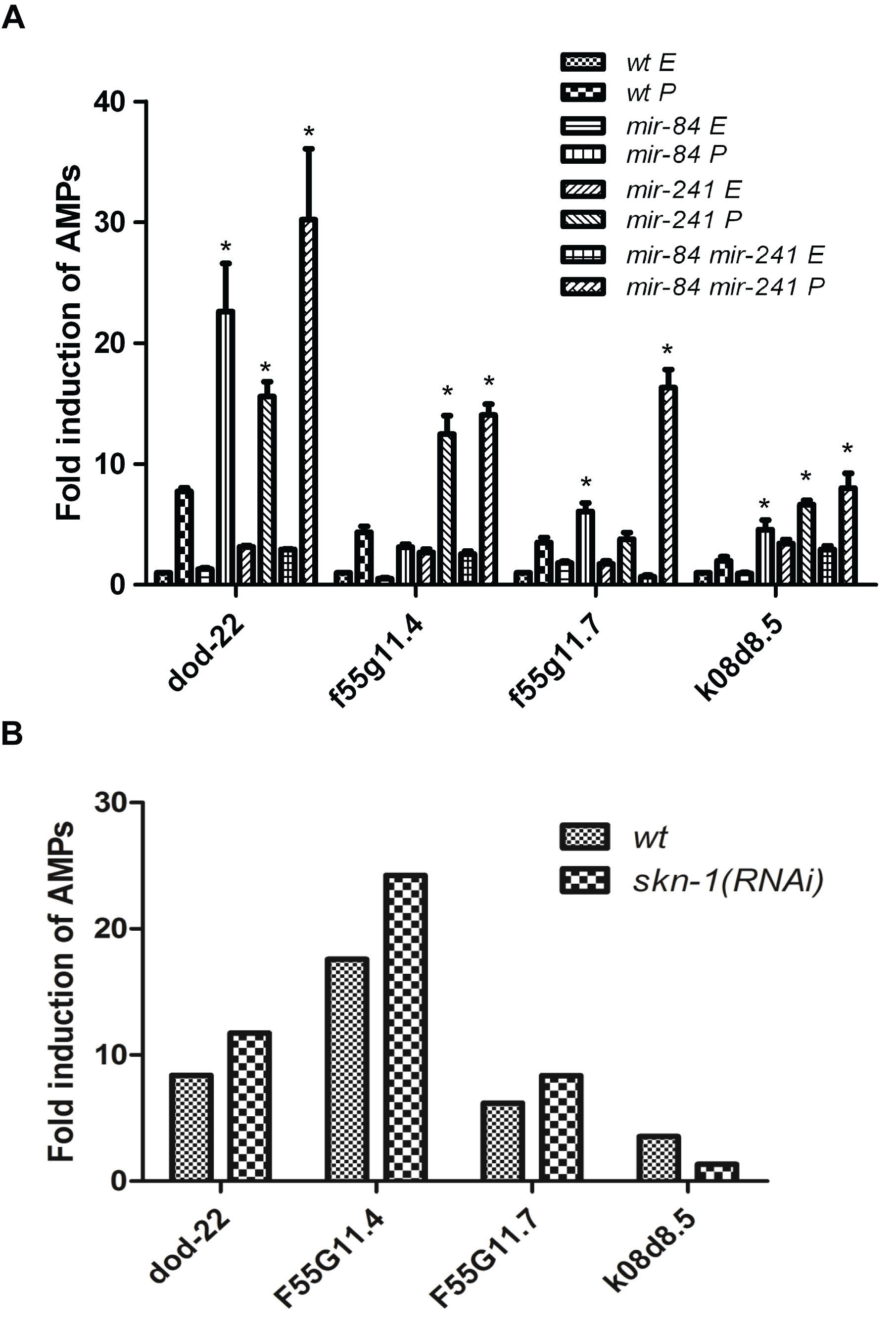

Supplement: Figure S9 — MiRNAs let-7s and SKN-1 regulate DAF-12-mediated AMPs expression. (A) Quantitative real-time PCR assay of antimicrobial gene expression of wild-type N2, mir-84(n4037) and mir-241(n4316) young adults fed E. coli or P. aeruginosa for 24 hours. (B) Quantitative real-time PCR assay of antimicrobial gene expression of wild-type N2 and skn-1(RNAi) young adults fed E. coli or P. aeruginosa for 24 hours. (TIF) [file ppat.1003545.s009.tif]

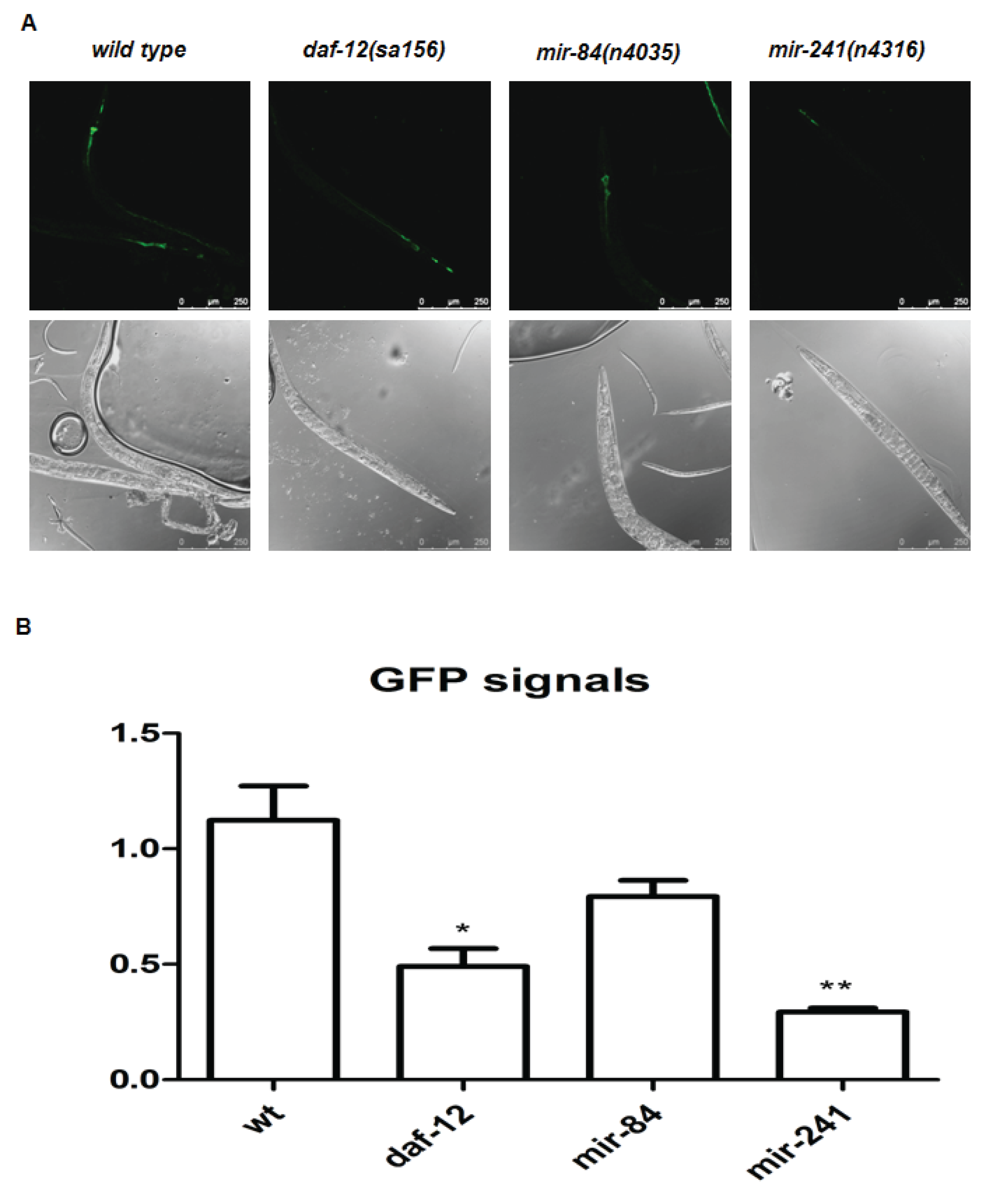

Supplement: Figure S10 — DAF-12 and let-7s miRNAs regulate bacterial accumulation in worm intestine. (A) Confocal imaging of wild-type N2, daf-12(sa156), mir-84(n4037) and mir-241(n4316) animals fed GFP-tagged P. aeruginosa for 24 hours. (B) Quantification of GFP signals in Supp. Fig. 10A. (TIF) [file ppat.1003545.s010.tif]

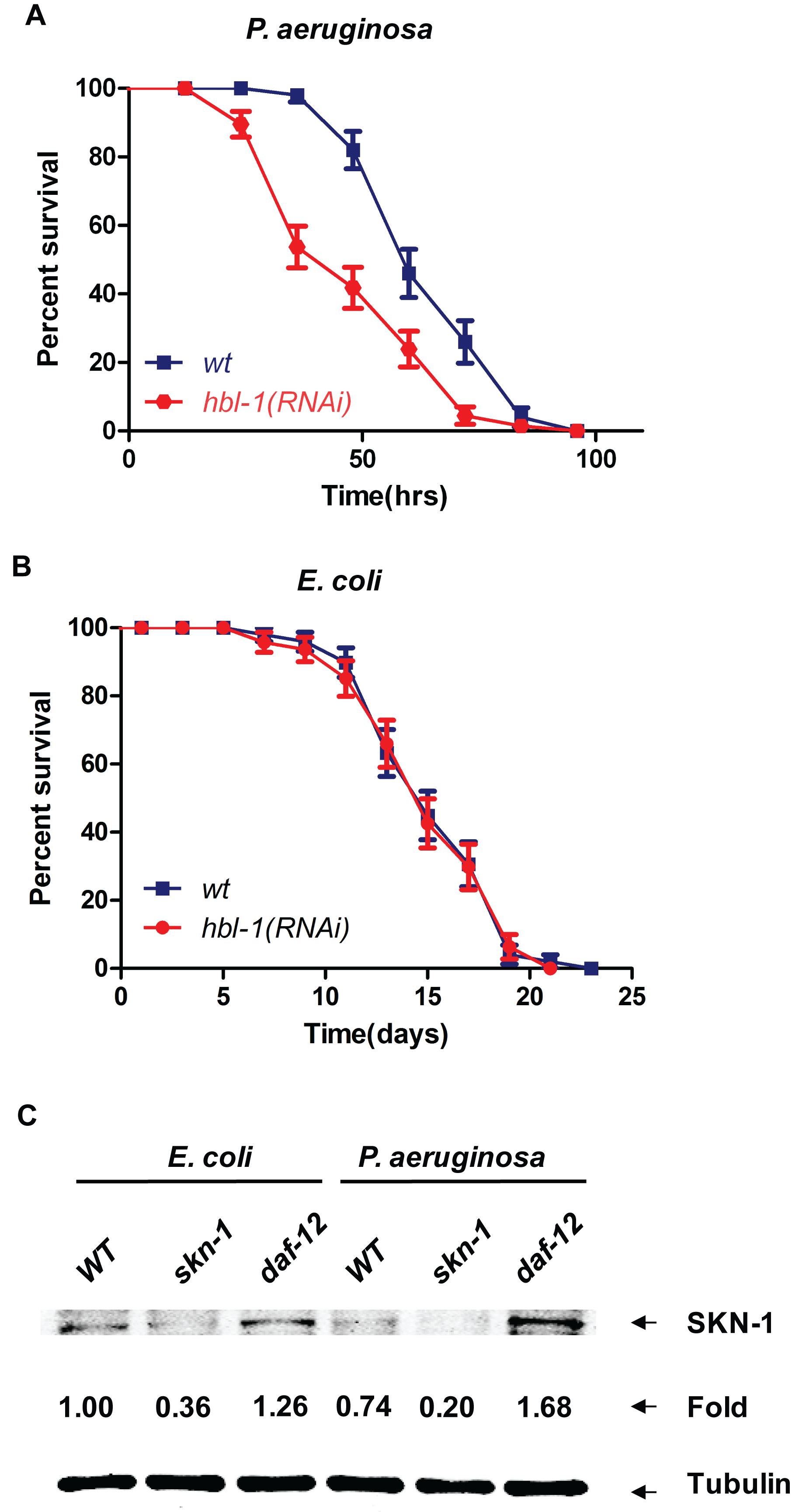

Supplement: Figure S11 — HBL-1 regulates innate immunity. (A) Survival curve of N2 and hbl-1(RNAi) (P<0.0001) worms on P. aeruginosa. (B) Lifespan assay of N2 and hbl-1(RNAi) (P = 0.8882) worms on E. coli. (C) Immunoblot analysis of the lysates from N2, skn-1(RNAi) and daf-12(rh61rh411) young adults fed E. coli or P. aeruginosa using anti-SKN-1 antibody and anti-tubulin antibody (loading control). (TIF) [file ppat.1003545.s011.tif]

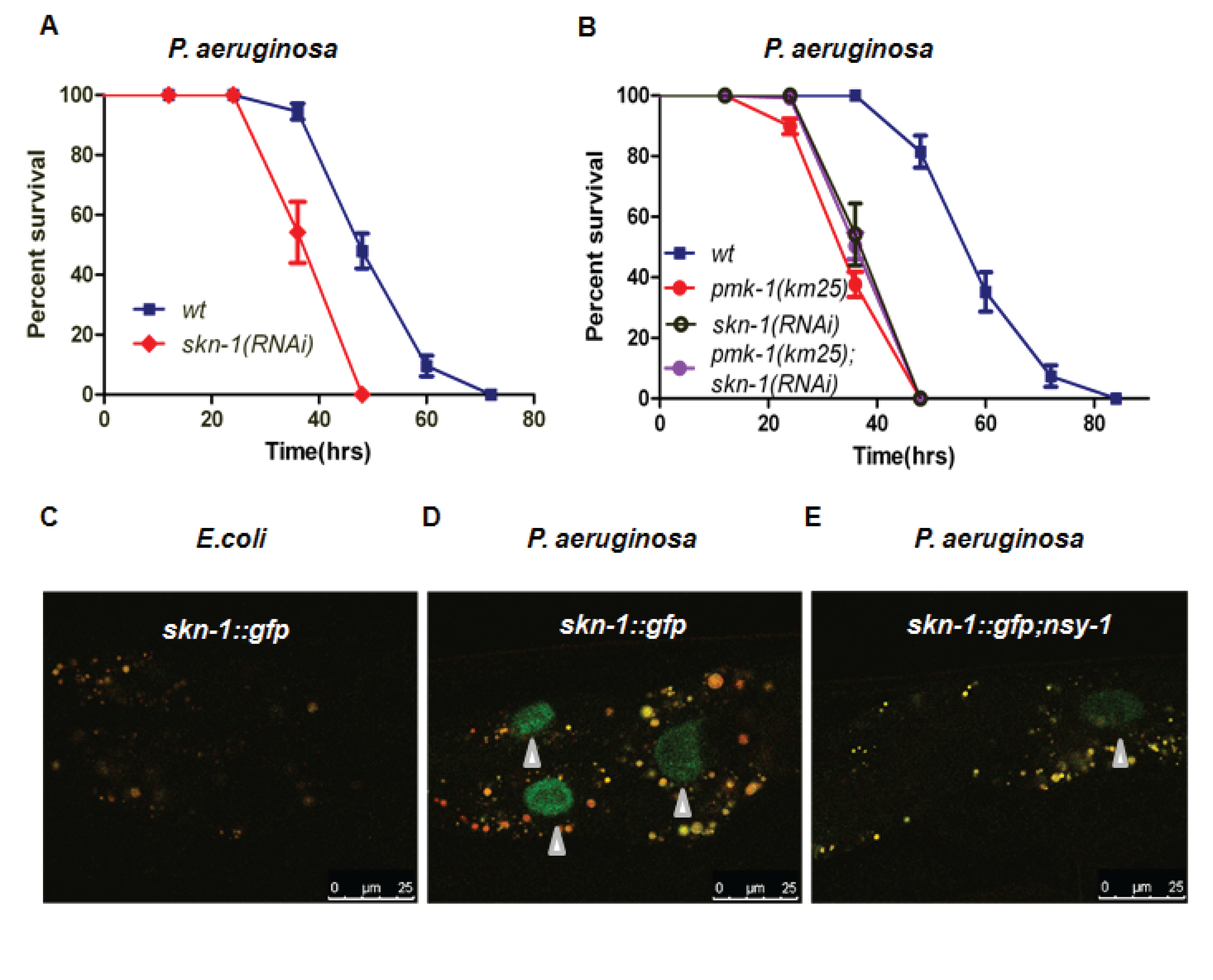

Supplement: Figure S12 — SKN-1 acts at downstream of PMK-1 to regulate innate immunity. (A) Survival curve of N2 and skn-1(RNAi) (P<0.0001) worms on P. aeruginosa. (B) Survival curve of wild-type N2, pmk-1(km25) (P<0.0001) and pmk-1(km25);skn-1(RNAi) (P = 0.0067) worms on P. aeruginosa. (C–E) Confocal microscopy of nsy-1 RNAi-treated or control RNAi-treated skn-1::GFP worms on P. aeruginosa or E. coli. Arrows shows the nuclear skn-1::GFP. (TIF) [file ppat.1003545.s012.tif]

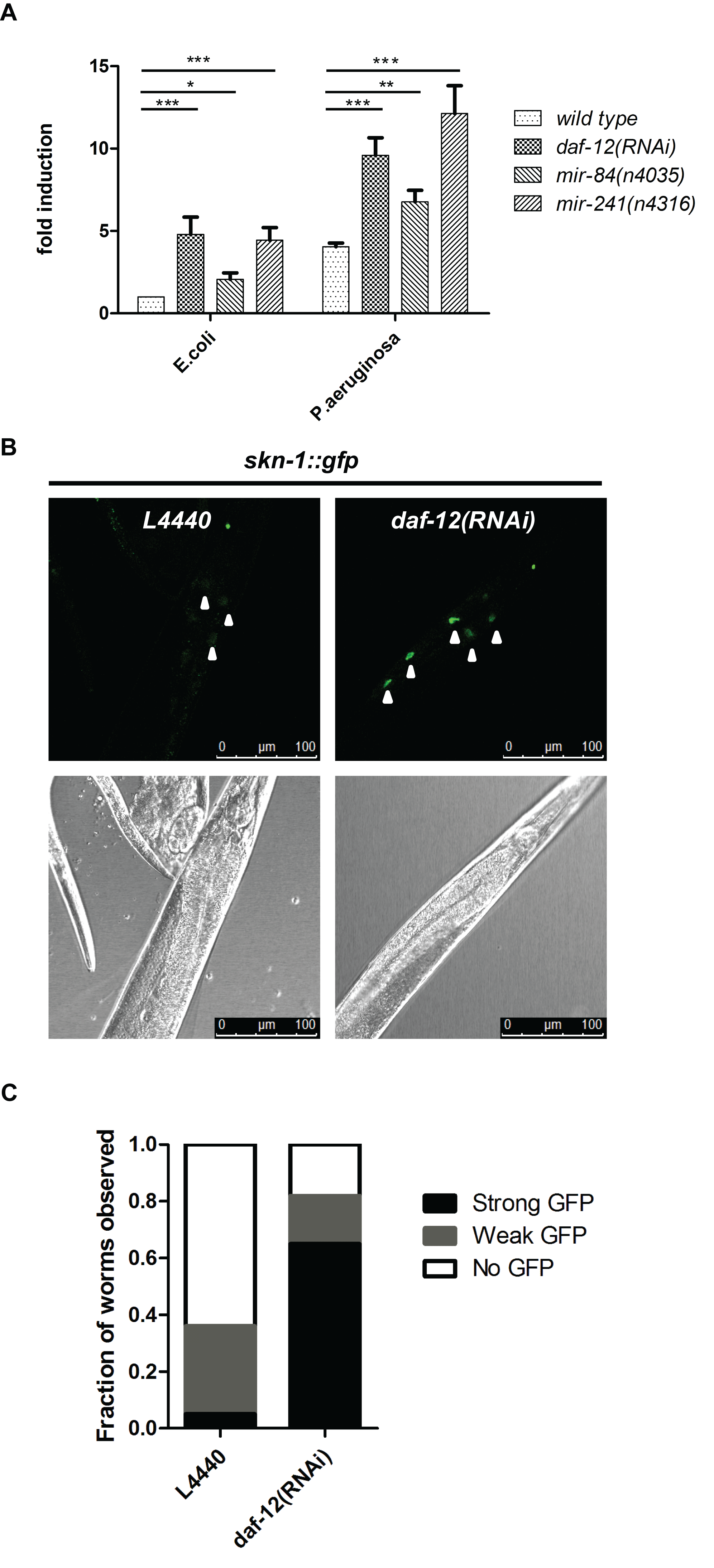

Supplement: Figure S13 — DAF-12 regulates SKN-1 activity. (A) Quantitative real-time PCR assay of gcs-1 expression of wild-type N2, daf-12(RNAi), mir-84(n4037) and mir-241(n4316) young adults fed E. coli or P. aeruginosa for 24 hours. (B) Confocal imaging of daf-12 RNAi treated or control treated young adults of skn-1::gfp transgenic worms. Arrows shows the nuclear skn-1::GFP. (C) Quantification of skn-1::gfp observed in worms treated with daf-12 RNAi (n = 23) or control (n = 19) in Fig. S13B. (TIF) [file ppat.1003545.s013.tif]

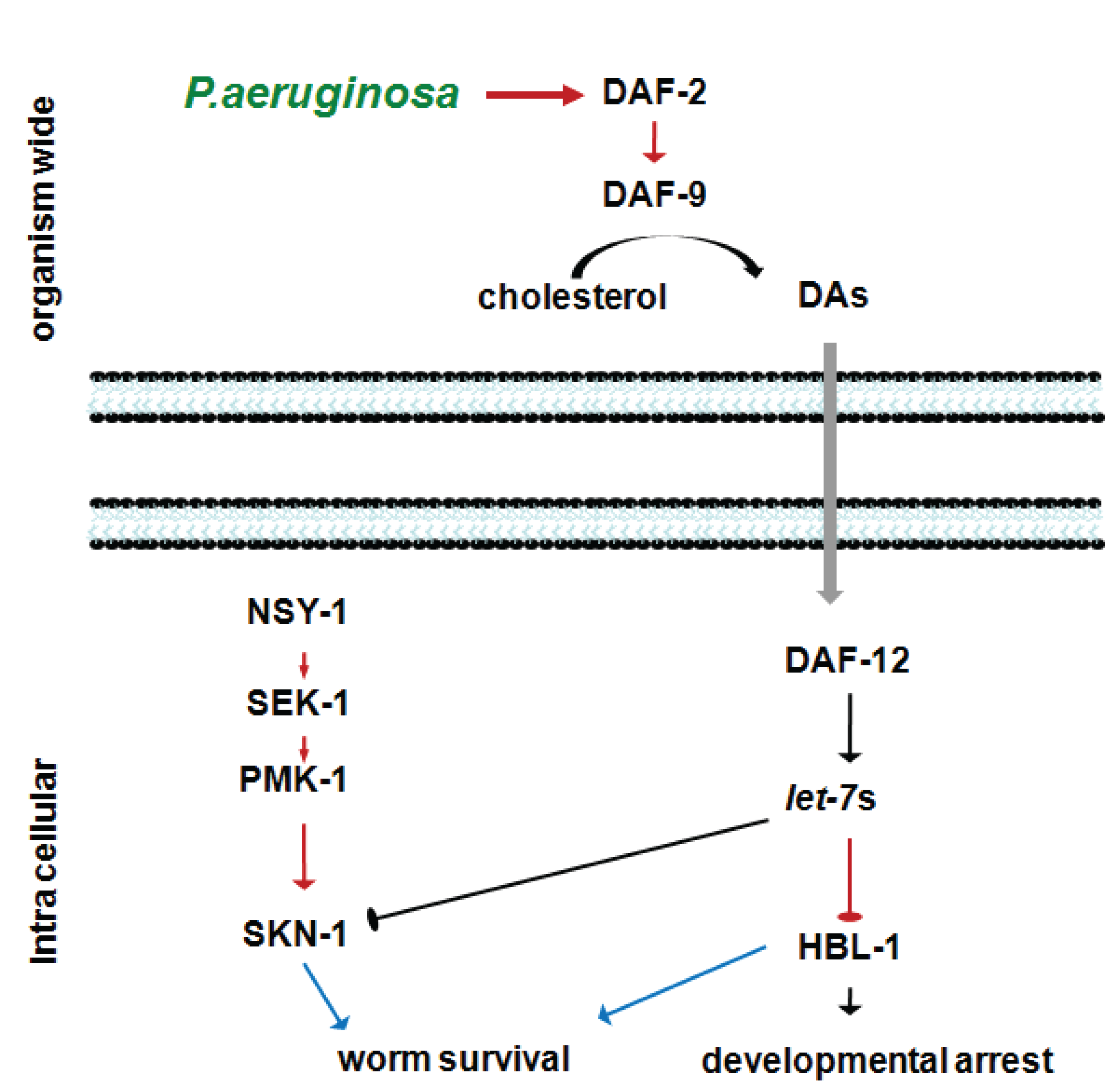

Supplement: Figure S14 — The hypothesized diagram. Caenorhabditis elegans nuclear receptor DAF-12 negatively regulates the pathogenic defense via its downstream microRNAs, let-7s, which may directly target SKN-1, thus counteract the activation of SKN-1 by NSY-1/PMK-1 pathway. (TIF) [file ppat.1003545.s014.tif]

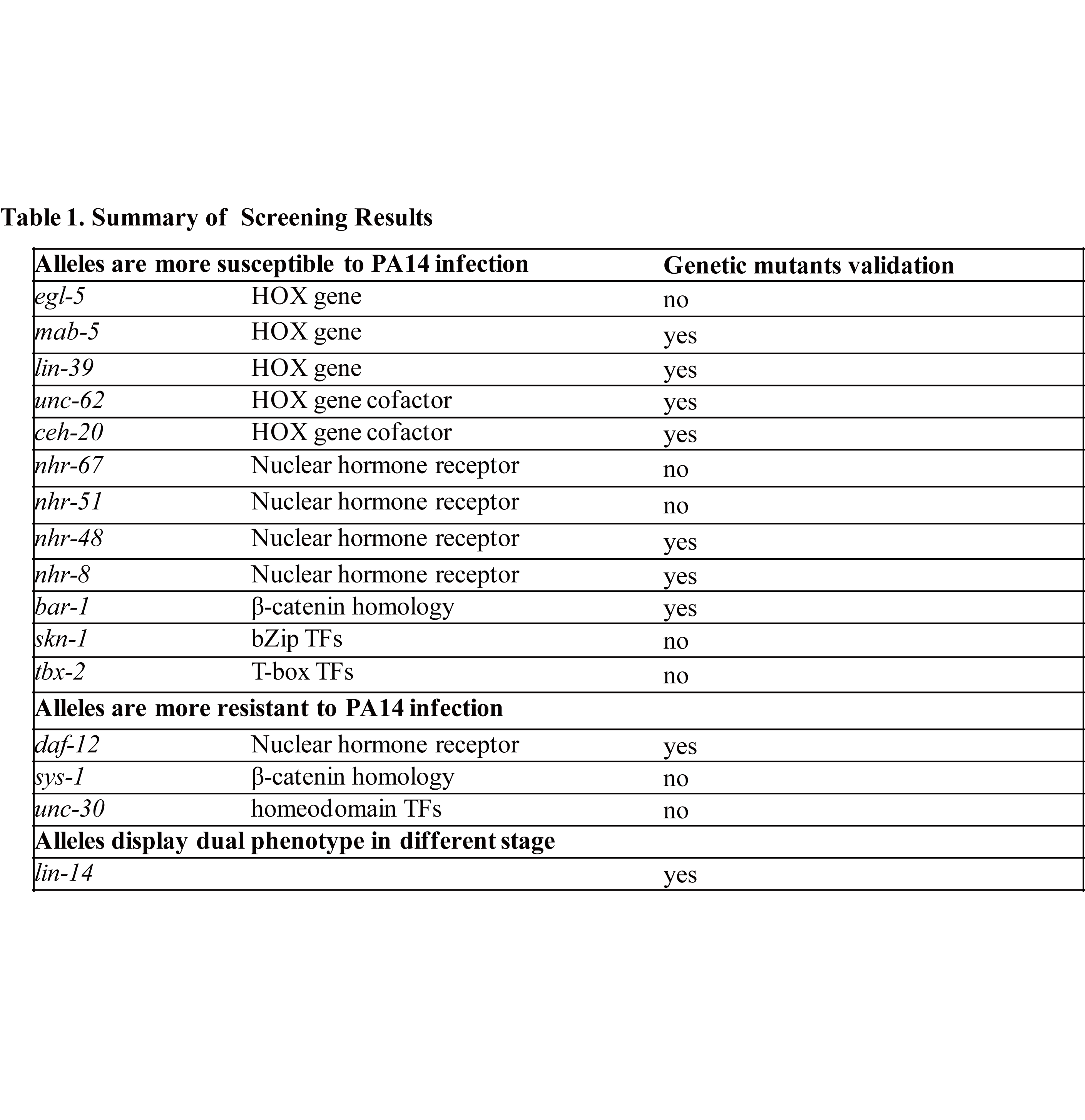

Supplement: Table S1 — List of alleles isolated from the RNAi screening. (TIF) [file ppat.1003545.s015.tif]

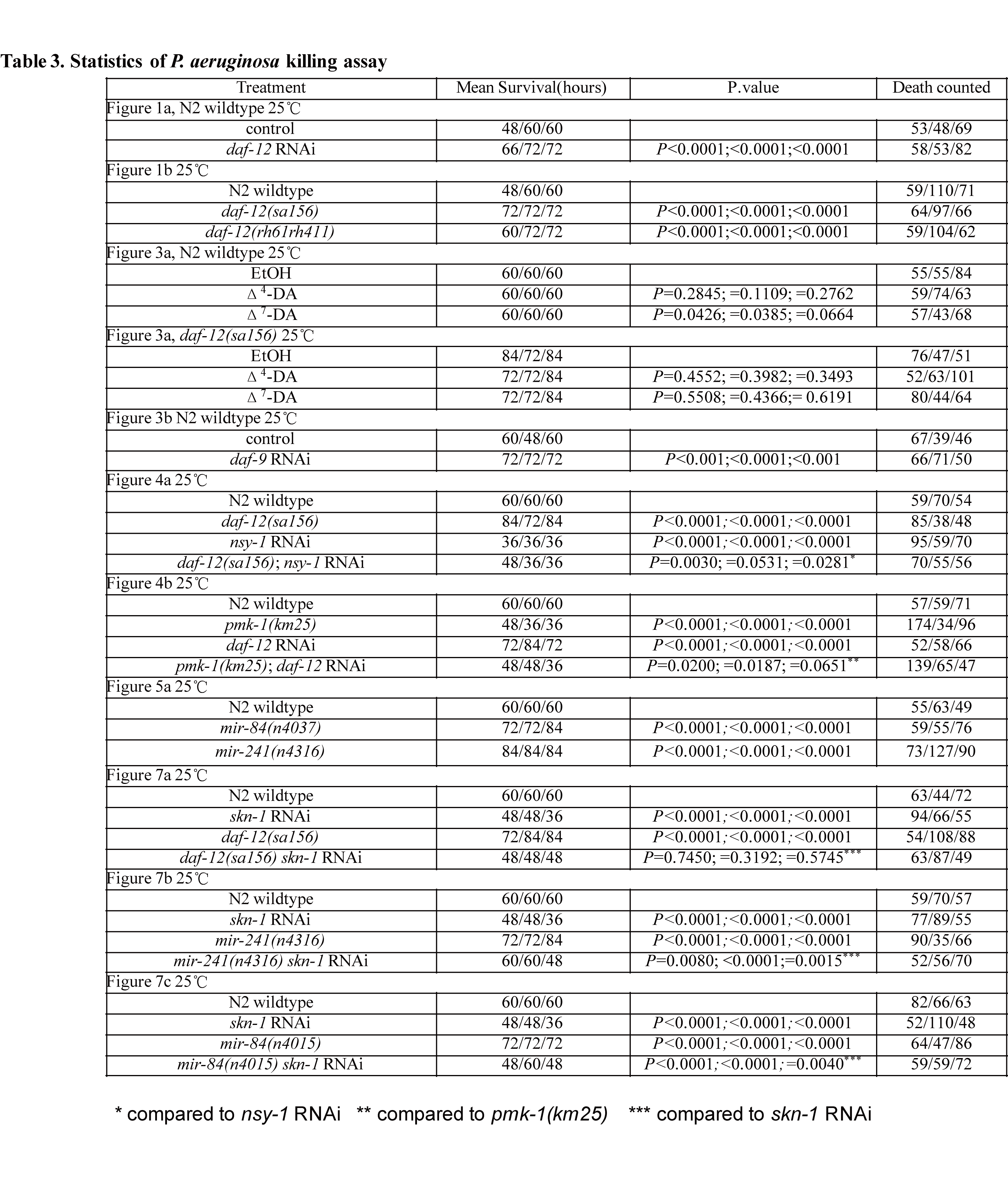

Supplement: Table S3 — The statistical analysis of all P. aeruginosa killing assays shown in figures. (TIF) [file ppat.1003545.s017.tif]

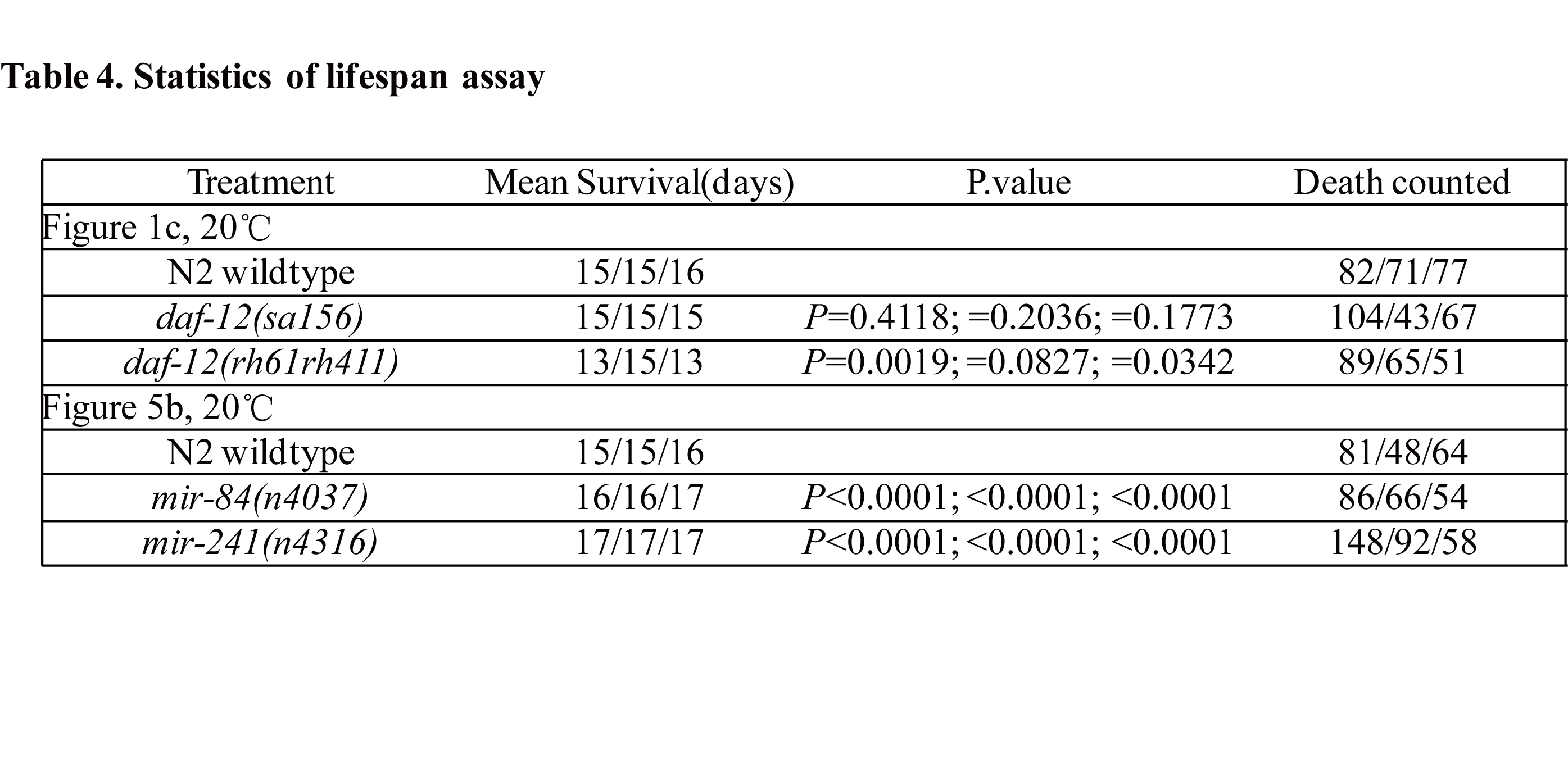

Supplement: Table S4 — The statistical analysis of all lifespan assays shown in figures. (TIF) [file ppat.1003545.s018.tif]

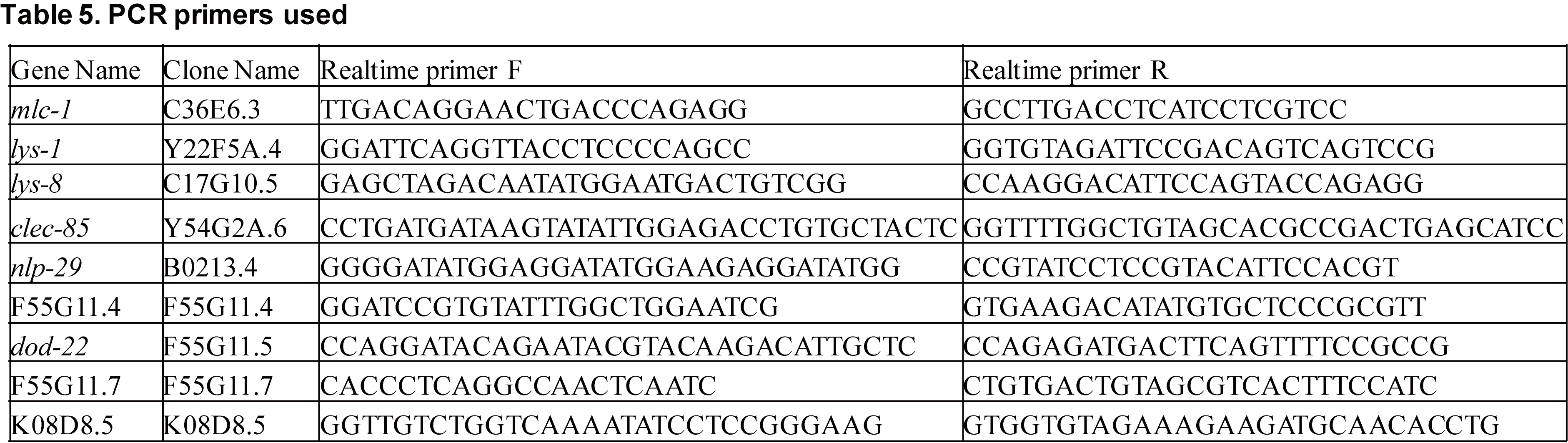

Supplement: Table S5 — The combination of primers used in quantitative real-time RT-PCR assay. (TIF) [file ppat.1003545.s019.tif]

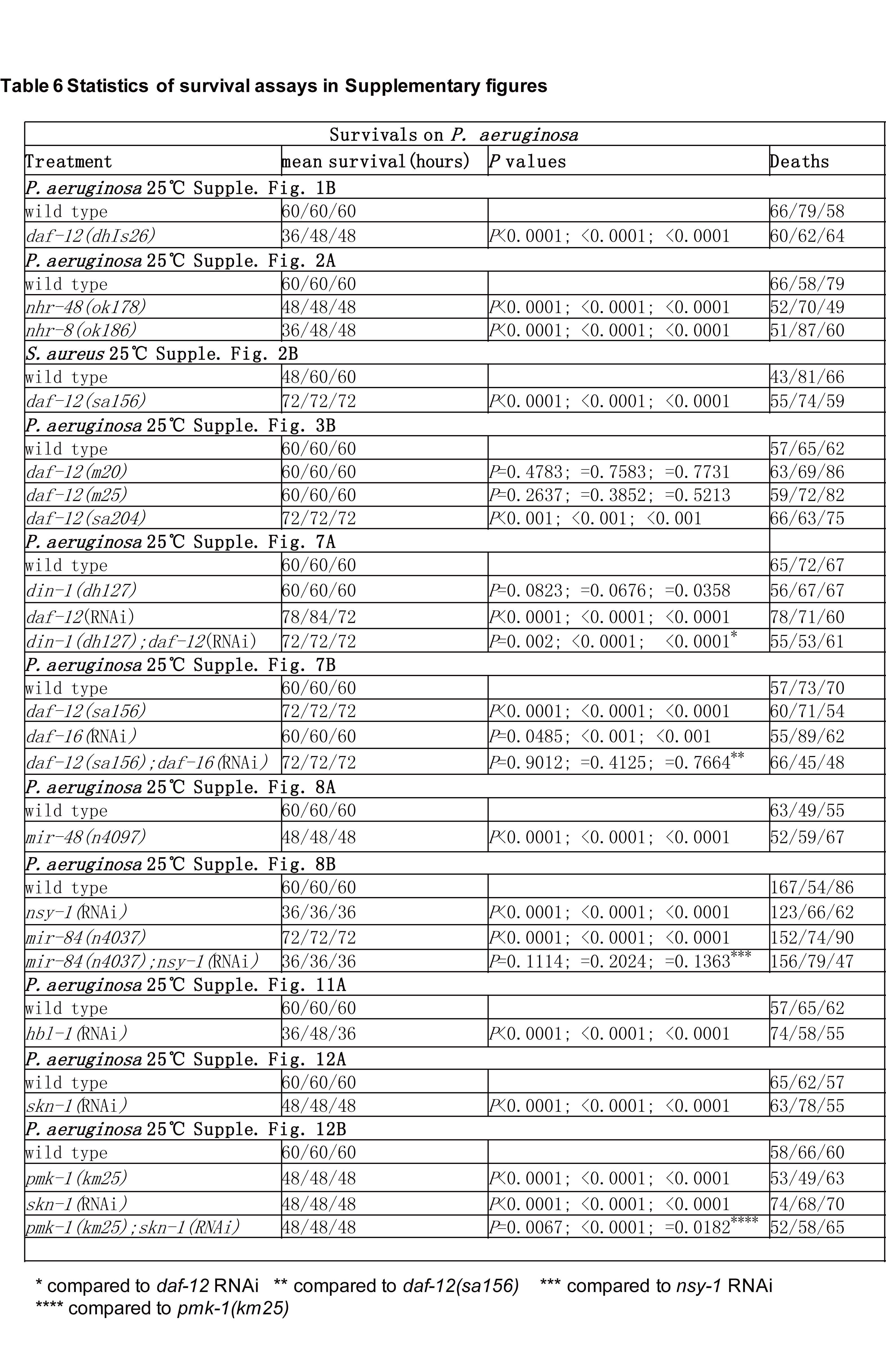

Supplement: Table S6 — The statistical analysis of all P. aeruginosa killing assays shown in supplementary figures. (TIF) [file ppat.1003545.s020.tif]

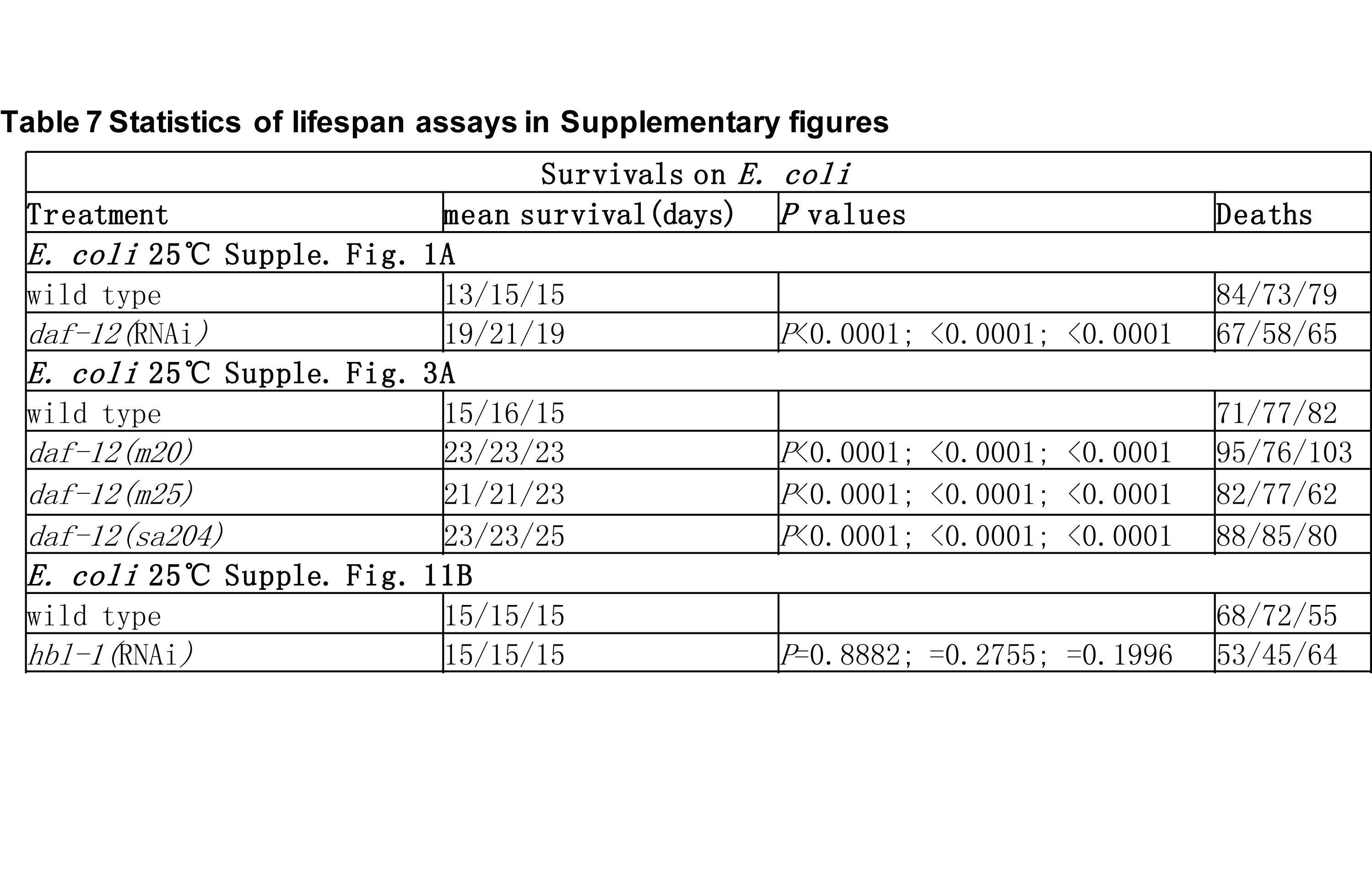

Supplement: Table S7 — The statistical analysis of all lifespan assays shown in supplementary figures. (TIF) [file ppat.1003545.s021.tif]
